# The role of biocatalysis in the asymmetric synthesis of alkaloids

**DOI:** 10.1039/c3ra42123f

**Published:** 2013-08-07

**Authors:** Joerg H. Schrittwieser, Verena Resch

**Affiliations:** a Department of Biotechnology , Delft University of Technology , Julianalaan 136 , 2628 BL Delft , The Netherlands . Email: j.schrittwieser@tudelft.nl ; Email: v.a.resch@tudelft.nl ; Fax: +31 152 781415 ; Tel: +31 152 782683

## Abstract

Alkaloids are not only one of the most intensively studied classes of natural products, their wide spectrum of pharmacological activities also makes them indispensable drug ingredients in both traditional and modern medicine. Among the methods for their production, biotechnological approaches are gaining importance, and biocatalysis has emerged as an essential tool in this context. A number of chemo-enzymatic strategies for alkaloid synthesis have been developed over the years, in which the biotransformations nowadays take an increasingly ‘central’ role. This review summarises different applications of biocatalysis in the asymmetric synthesis of alkaloids and discusses how recent developments and novel enzymes render innovative and efficient chemo-enzymatic production routes possible.

## Abstract 2

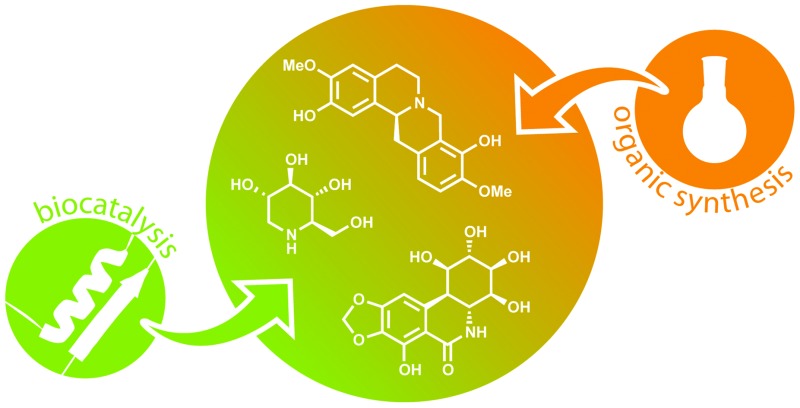
This article reviews the progress in chemo-enzymatic alkaloid synthesis over the last 25 years, focusing on recent developments that have led to significant improvements in terms of step-economy and yield.

## 1. Introduction

The alkaloids are a structurally diverse group of nitrogen-containing secondary metabolites with a relatively wide distribution in nature and a broad range of biological activities.^1^ While the original definition of the term ‘alkaloid’, introduced by the German pharmacist Carl F. W. Meissner in 1819,^2^ covers only plant-derived substances with alkaline character, modern conceptions are usually much broader. For instance, S. William Pelletier proposed a very simple definition in 1983, saying that “*An alkaloid is a cyclic organic compound containing nitrogen in a negative oxidation state which is of limited distribution among living organisms*” .^1d,3^ The total number of known structures that can be categorised as alkaloids according to these general criteria exceeds 20,000,^4^ and around 100 new compounds are still discovered annually.^1*b*^ The higher plants are a major source of alkaloids, as approximately 25% of all plant species contain these constituents,^1*b*^ but their occurrence in animals, insects, microorganisms, and lower plants is also well documented.^1*d*^

The natural functions of plant alkaloids are thought to be mainly associated with the interactions of the plant with other organisms–for instance, protection against herbivores or pathogens, or attraction of pollinating insects, seed dispensing animals or root nodule bacteria.^1d,5^ Hence, it is not surprising that many alkaloids exert potent pharmacological activities, a fact that has been recognised by humans and exploited for both therapeutic and recreational purposes for thousands of years.^1c,6^ Also in modern medicine, many alkaloids and their derivatives have found application as drugs and even more are currently under investigation in clinical trials (for a selection of therapeutically important alkaloids, see Table 1). Estimated global sales of over 4 billion US$ (2002) for alkaloids applied in medicine illustrate both the interest in this substance class and the necessity for efficient production routes.^7^ However, not all alkaloids of public demand can be isolated from their natural source in sufficient quantities, and even if isolation is possible on large scale, it is often a very expensive and time-consuming procedure.^7^ Especially for minor constituents the purification effort to arrive at a chemically well-defined product can be enormous, and isolation yields are typically low.

**Table 1:** Examples of alkaloids with medicinal application^*a*^

Alkaloid (active ingredient)	Natural source^1*d*^	Therapeutic use
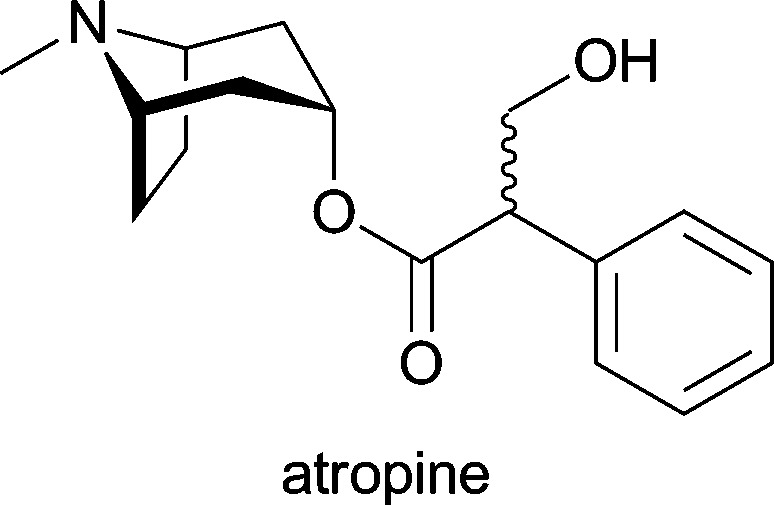	*Atropa belladonna*; industrially isolated from *Duboisia* species, *Hyoscyamus muticus* and *Hyoscyamus niger*	antispasmodic; treatment of organo-phosphate poisoning; against myasthenia gravis; against arrhythmias; local treatment for muscular rheumatism, sciatica and neuralgia; in ophthalmology as mydriatic and cycloplegic drug^1*d*^
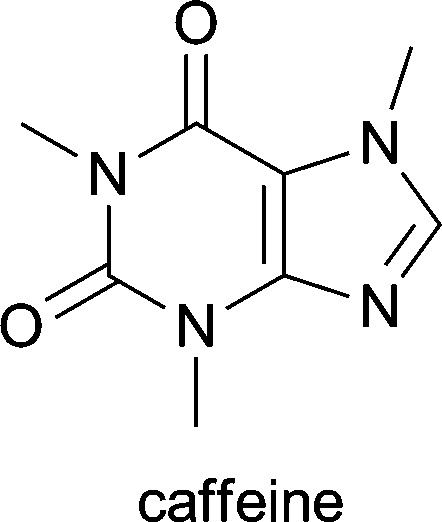	*Caffea arabica*, *Paullinia cupana*, *Cola nitita* and *Cola acuminata*	added to analgesics to increase their activity; used in the treatment of neonatal apnoea and atopic dermatitis^1*d*^
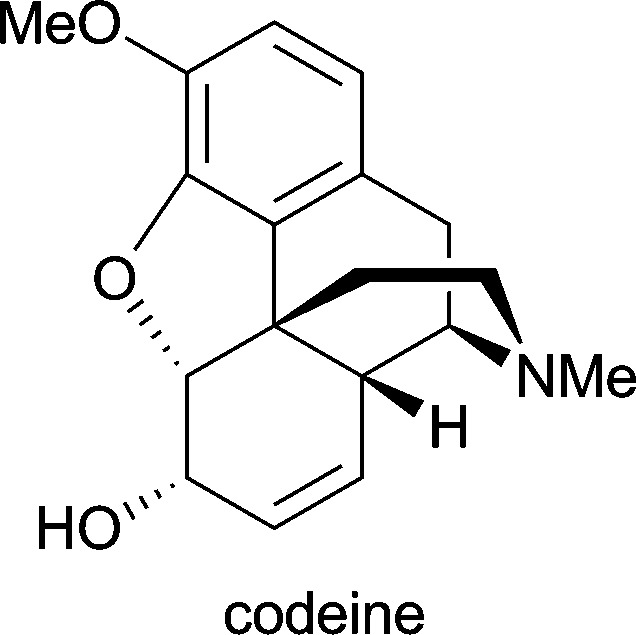	*Papaver somniferum*	as antitussive in treatment of cough; as analgesic and mild sedative^1*d*^
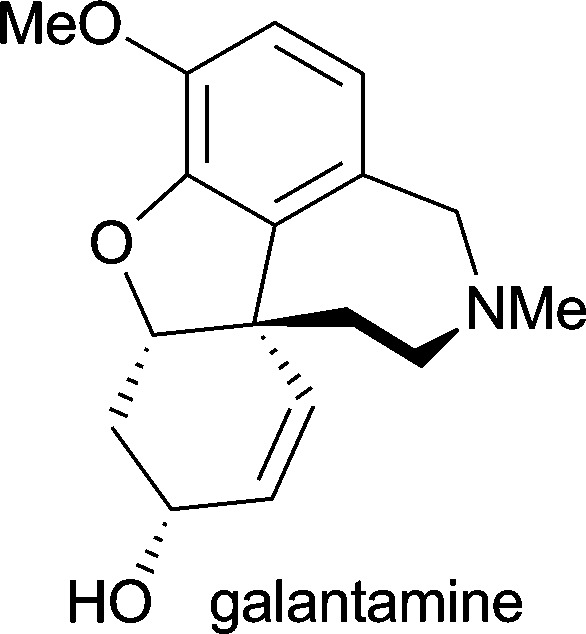	*Galanthus caucasicus*, *Galanthus woronowii* and *Galanthus nivalis*	in the treatment of Alzheimer's disease^24^
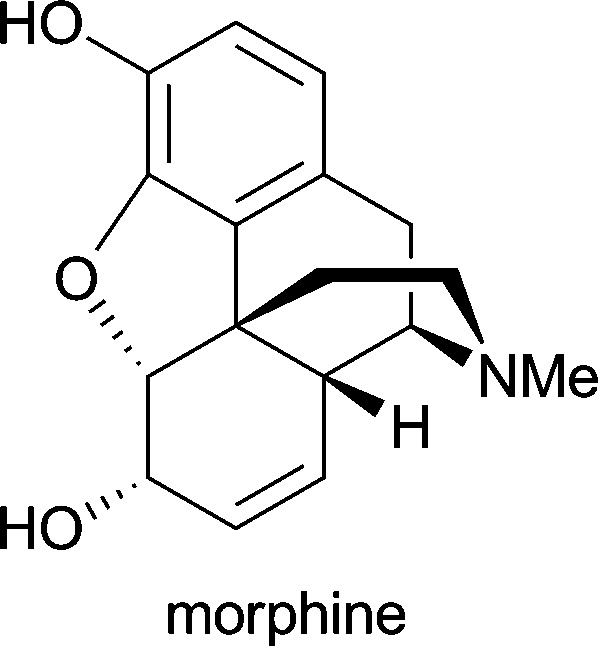	*Papaver somniferum*	pain control; treatment of diarrhoea^1*d*^
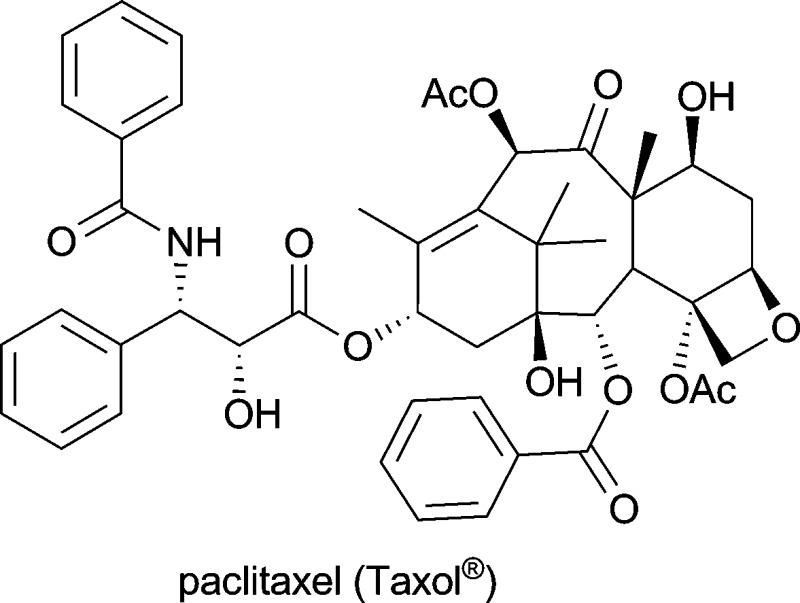	*Taxus brevifolia*; produced industrially by *Taxus* plant cell fermentation	in treatment of mamma and ovary carcinoma and several other malignancies^1*d*^
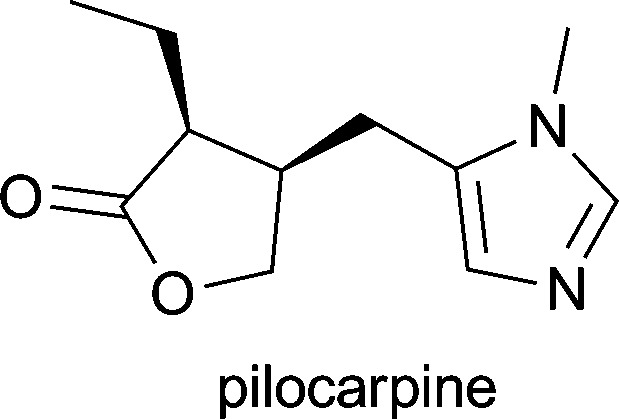	*Pilocarpus jaborandi*, *Pilocarpus pennatifolius*, *Pilocarpus racemosus* and *Pilocarpus microphyllus*	in treatment of open-angle glaucoma^1*d*^
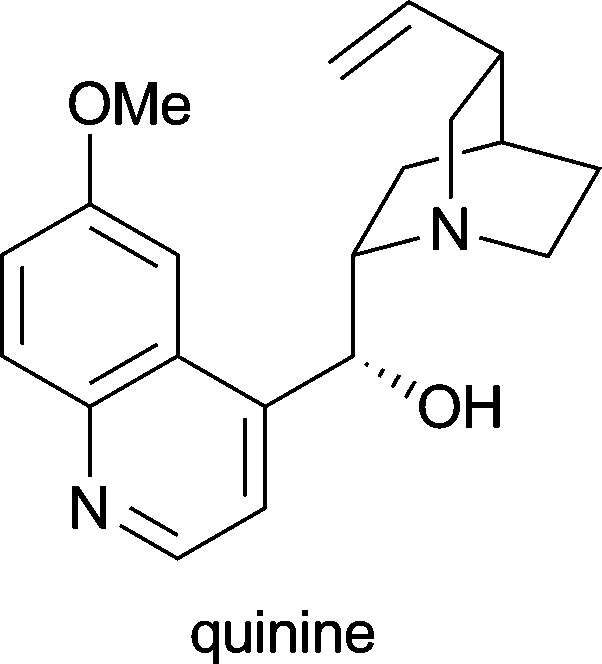	*Cinchona succirubra*	in treatment of malaria, babesiosis and myotonic disorders^1*d*^
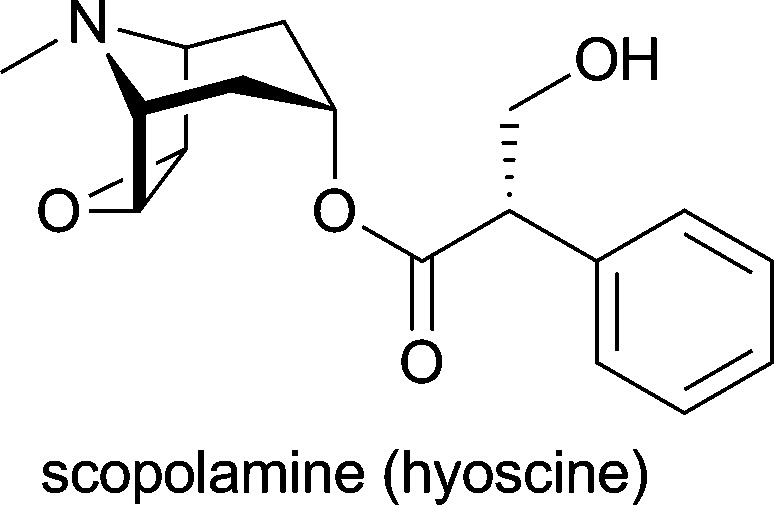	*Duboisia myoporoides*, *Duboisia leichhardtii*	against acute motion sickness; as premedicant before induction of general anaesthesia^1*d*^
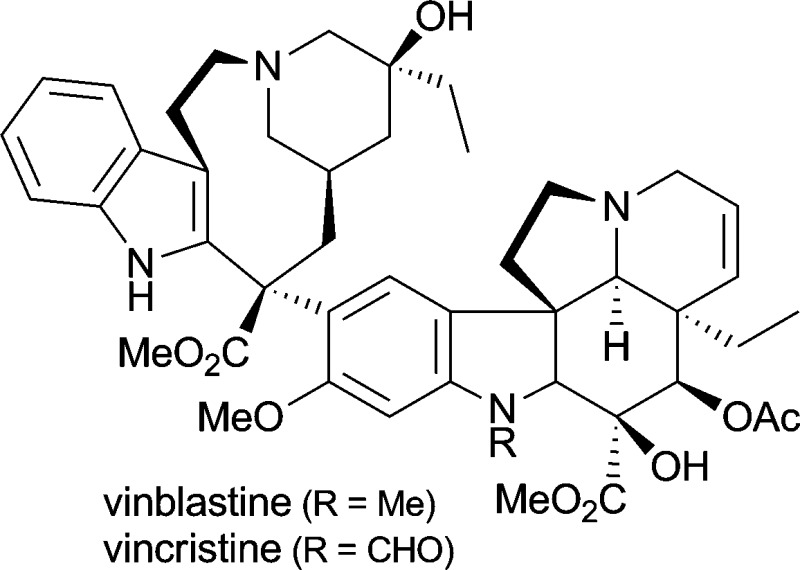	*Catharanthus roseus*	in treatment of Hodgkin's disease and other lymphoma, testicular cancer and a variety of solid neoplasms^1d,5a^

^*a*^Apart from galantamine, all listed substances are part of the World Health Organization's *Model List of Essential Medicines* (17th ed., for adults).^25^

On the other hand, the intricate molecular architecture of many alkaloids–with diverse functional groups and often multiple stereogenic centres–also makes them challenging targets for total synthesis. In view of these difficulties it is just the more impressive that many complex alkaloids have indeed been synthesised in the lab; however, long sequences involving extensive protective group chemistry are often required, resulting in very limited overall yields. Consequently, the transfer of these synthetic routes to production scale usually represents a major problem. An excellent example for this discrepancy–discussed in a recent account by Walji & MacMillan^8^–is the case of paclitaxel (Taxol®): Six ingenious and highly acclaimed total syntheses of this complex alkaloid (for the structure, see Table 1) have been developed to date, the most efficient of which gives the target compound in 0.4% yield over 37 steps.^9^ Unfortunately, large-scale production of paclitaxel by this route is not practical or economically feasible, and neither is its isolation from natural sources. Today, the growing demand in this natural anti-tumour agent is met by plant cell fermentation,^10^ and the environmental benefits of this production method have been recognised with the 2004 *Presidential Green Chemistry Award*.^11^

The paclitaxel example already illustrates that biological methods can represent favourable options for alkaloid production. Indeed, biotechnology in its broadest sense offers several strategies that may help to overcome some of the above-mentioned supply limitations. For instance, genetic modification of alkaloid-containing plants can be used to increase production levels and hence facilitate isolation.^12^ Plant cell fermentation can be used to achieve production of alkaloids by the native source organism under controlled and scalable conditions. Entire secondary metabolic pathways can be engineered into suitable microbial hosts, which thus become able to biosynthesise the corresponding natural products.^13^ This approach has recently been applied to the fermentative production of the benzylisoquinoline alkaloid (*S*)-reticuline from simple carbon sources such as glycerol or glucose, using an engineered *E. coli* strain.^14^ In a related study, (*S*)-reticuline has been obtained by fermentation from dopamine, and has been further transformed into aporphine or protoberberine alkaloids using transgenic *Saccharomyces cerevisiae*.^15^ The implementation of an entire biosynthetic pathway for the production of benzylisoquinolines into *Saccharomyces cerevisiae* has also been reported.^13,16^

In more chemistry-oriented settings, biocatalysis can be employed for the selective modification of alkaloids to access non-natural or less abundant derivatives. A classical example is the use of the enzymes morphine dehydrogenase and morphinone reductase from *Pseudomonas putida* M10 for the interconversion of morphinan alkaloids.^17^ More recent examples include the laccase-catalysed coupling of catharanthine and vindoline to yield anhydrovinblastine,^18^ the laccase-catalysed hydroxylation of ergot alkaloids,^19^ or the coupling of the alkaloid sinomenine with electron-rich arenes mediated by the fungi *Antrodiella semisupina* ^20^ and *Coriolus unicolor*.^21^ Finally, biocatalytic methods can be employed in the asymmetric total synthesis of alkaloids, thereby combining the flexibility of chemical routes with the high degree of chemo- and enantioselectivity offered by enzymes. This interdisciplinary approach seems to be particularly fruitful, as a considerable number (approx. 150) of chemo-enzymatic total syntheses of alkaloids have been reported over the last 25 years, which in many cases prove more efficient than either a purely chemical synthesis or isolation of the target compound from natural sources.

Three main strategies for chemo-enzymatic alkaloid synthesis can be distinguished: (1) the biocatalytic preparation of chiral building blocks, which are chemically transformed into the target compounds, (2) the biocatalytic kinetic resolution, desymmetrisation or deracemisation of alkaloids that have been synthesised by chemical means, and (3) the chemo-enzymatic synthesis of alkaloids *via* biocatalytic C–N and/or C–C bond formation in the asymmetric key step. The first approach is definitely the most versatile, and also by far the most explored, but in recent years biocatalytic deracemisation and asymmetric biocatalytic C–C bond formation have gained momentum. This review provides an overview of all three above-mentioned strategies and discusses recent developments that are likely to change the role of biocatalysis in future alkaloid synthesis.

## 2. Biocatalytic asymmetric synthesis of chiral building blocks

The chiral building block (also called ‘chiral synthon’ or ‘chiron’) approach to asymmetric synthesis involves the recognition of structural elements in the target molecule that can be traced back retrosynthetically to readily available chiral molecules.^22^ Classically, the latter are derived from the ‘chiral pool’ of amino acids, carbohydrates, terpenes, *etc.*, while in chemo-enzymatic approaches the chiral building blocks are compounds that can be obtained in high enantiomeric purity by a biocatalytic reaction.^23^ In the context of alkaloid chemistry, lipases and esterases as well as toluene dioxygenase are most frequently used for the generation of chiral building blocks, and a large number of structurally diverse alkaloids have been synthesised using these enzymes.

### 2.1 Lipases and esterases

Their excellent stereoselectivity and broad substrate scope, their ability to work in organic solvents, and also their broad commercial availability have made lipases and esterases the most widely used biocatalysts in organic synthesis. Therefore, it is not surprising that also the majority of chemo-enzymatic syntheses of alkaloids rely on these enzymes. When applied in a kinetic resolution, they can provide access to both enantiomers of a chiral building block in 50% maximum yield. However, desymmetrisation of *meso*-compounds–which does not suffer from this limitation–is common as well. This strategy can also afford both enantiomers of the product, provided that stereocomplementary enzymes are available, or that the desymmetrisation can be run in both hydrolytic and acylative direction (*cf.* Scheme 2). Both reaction types have been used to prepare a considerable number of chiral building blocks, most of which can be categorised structurally into the groups of cyclic alcohols, piperidine derivatives, and nitrones.

#### 2.1.1 Cyclic alcohol building blocks

**Scheme 1:**
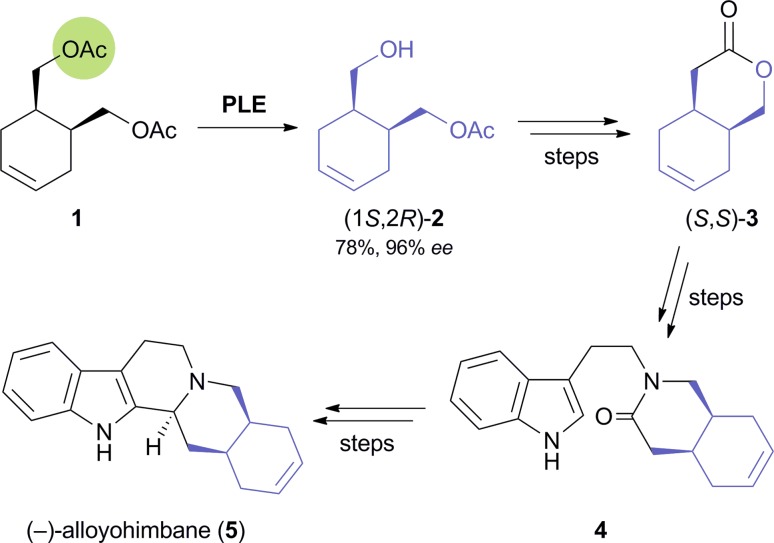
Chemo-enzymatic synthesis of (–)-alloyohimbane (**5**).^28^

The earliest report on the application of a biocatalytically obtained building block in the asymmetric total synthesis of an alkaloid is a brief communication by Renata Riva and co-workers published in 1987, which describes the preparation of (–)-alloyohimbane (**5**) from the hydroxy ester (1*S*,2*R*)-**2** (Scheme 1).^26^ This building block was obtained in 96% *ee* by hydrolytic desymmetrisation^27^ of the corresponding *meso*-diacetate **1** using pig liver esterase (PLE) as biocatalyst. The hydroxy ester was then converted into the lactone (*S*,*S*)-**3**, which was coupled with tryptamine to give amide intermediate **4**, from which **5** was prepared following a literature procedure.

**Scheme 2:**
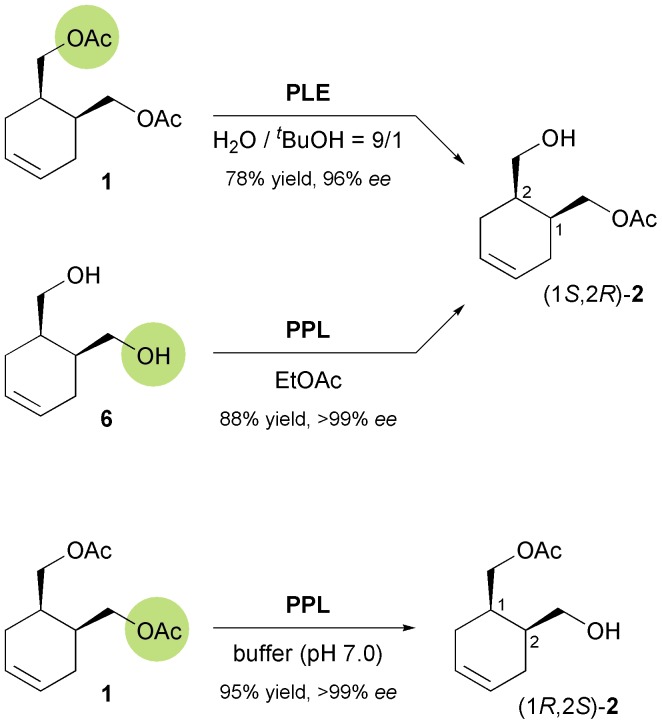
Options for the enantioselective preparation of building block **2** using hydrolases.

**Fig. 1:**
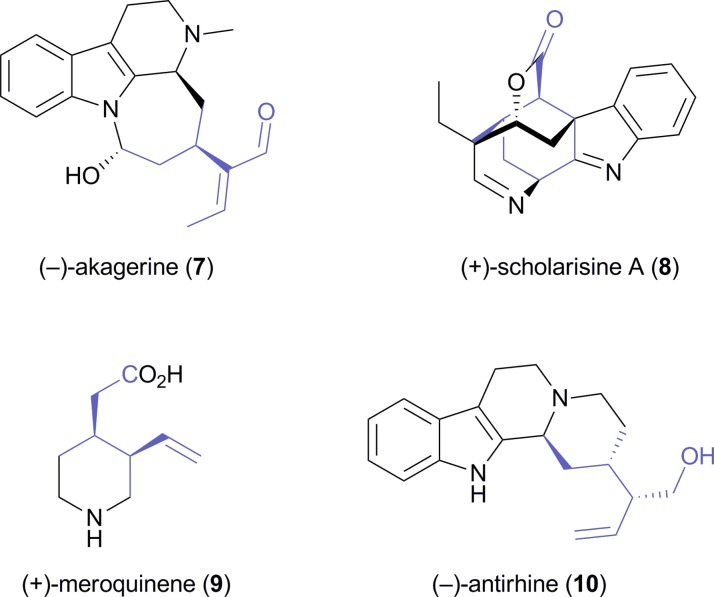
Alkaloids synthesised from the chiral building block **2**.

A later study found it difficult to reproduce the excellent optical purity of (1*S*,2*R*)-**2** in the PLE-catalysed hydrolysis process and moved to stereoselective monoacetylation of the corresponding *meso*-diol **6** (Scheme 2), employing porcine pancreatic lipase (PPL) in anhydrous ethyl acetate, to access the same compound in 88% yield and >99% *ee* on gram scale.^29^ The building block was again converted into (*S*,*S*)-**3**,^30^ which *via* 10 additional steps was further elaborated into the *Strychnos* alkaloid (–)-akagerine (**7**, Fig. 1), obtained in 19% overall yield from **2**. Very recently, (1*S*,2*R*)-**2** has been ‘rediscovered’ and used in the asymmetric total synthesis of the complex polycyclic indole alkaloid (+)-scholarisine A (**8**, Fig. 1), first isolated from the leaves of *Alstonia scholaris* in 2008. The target compound was obtained in 0.4% overall yield *via* 20 synthetic steps, which include a nitrile hydrogenation/epoxide aminolysis cascade, a Fischer-type indole synthesis, and an oxidative lactonisation.^31^

The opposite enantiomer of **2**, accessible *via* stereoselective monohydrolysis of **1** with PPL (Scheme 2) has also found application in the asymmetric synthesis of alkaloids: It has been used by Danieli and co-workers in the preparation of (+)-meroquinene (**9**), a degradation product of cinchonine and key intermediate in the synthesis of *Cinchona* alkaloids,^32^ and of the indole alkaloid (–)-antirhine (**10**, Fig. 1).^33^

**Scheme 3:**
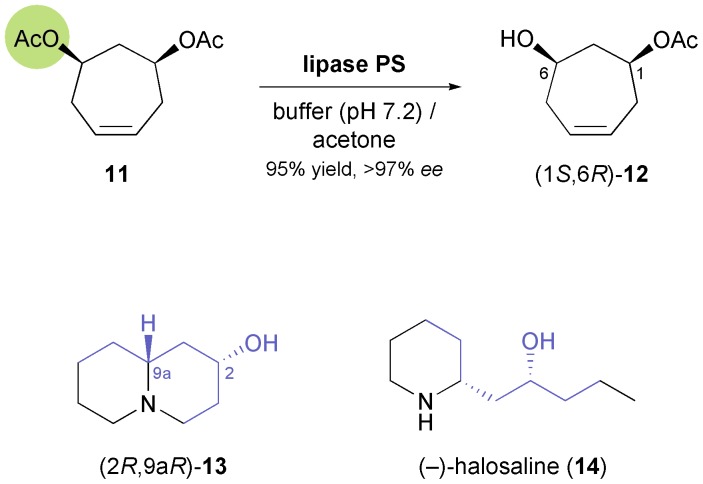
Desymmetrisation of *meso*-diacetate **11** using lipase PS affords compound **12**, a building block used in the synthesis of alkaloids **13** and **14**.

Lesma and co-workers, on the other hand, have extended the concept of hydrolytic desymmetrisation of *meso*-diacetates to the cycloheptene derivative **11**, obtaining the monoacetate (1*S*,6*R*)-**12** (Scheme 3) in 95% yield and >97% *ee* using lipase PS.^34,35^ From this building block, several 4-hydroxypiperidine derivatives such as *cis*-4-hydroxy-2-pipecolic acid, a hydroxylated quinolizidine **13**, and the piperidine alkaloid (–)-halosaline (**14**, Scheme 3) were prepared,^34,36^ the latter in a very elegant sequence involving ruthenium-catalysed ring-rearrangement metathesis as a key step.

**Scheme 4:**
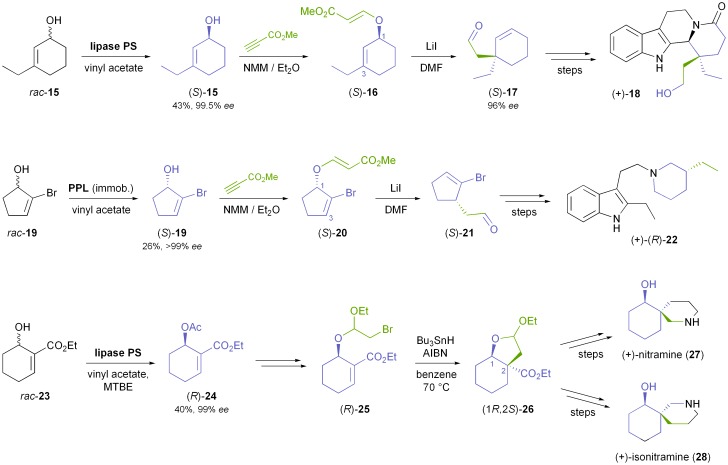
Use of enzymatically derived cycloalkenol building blocks in the synthesis of indole alkaloids and spirocyclic piperidine alkaloids.

Another common class of chiral building blocks in alkaloid synthesis are cyclic allylic alcohols with substituents on the double bond, such as 3-ethylcyclohexenol **15** (Scheme 4). The kinetic resolution of this and similar compounds with various lipases has been studied by Palmisano and co-workers,^37^ who also demonstrated the conversion of (*S*)-**15**, obtained from the racemate in 43% yield and 99.5% *ee* using *Burkholderia cepacia* lipase^35^ in neat vinyl acetate, into alkaloids of the eburnane type (*e.g.* **18**).^38^ A key step in their synthesis is a transfer of chirality from C1 of the cyclohexenol structure to C3 by means of a decarboxylative Claisen rearrangement, thus setting up a quaternary carbon chiral centre in excellent enantiomeric purity (*ee* = 96%). The authors followed a similar strategy in the asymmetric synthesis of (+)-decarbomethoxy-15,20;16,17-tetrahydrosecodine (**22**), where a Claisen rearrangement of the ester (*S*)-**20** was used to establish a tertiary carbon chiral centre (Scheme 4).^39^ Immobilised porcine pancreatic lipase (PPL) in neat vinyl acetate was used for the kinetic resolution of *rac*-**19** (the precursor to **20**), and the bromine atom in the substrate served the sole purpose of enabling a sufficiently high enantioselectivity (*E* = 133) in this reaction by increasing the steric demand of one side of the substrate. A ‘transfer of chirality’ concept was also applied by Yamane & Ogasawara in their chemo-enzymatic syntheses of spirocyclic piperidine alkaloids:^40^ Kinetic resolution of carbethoxycyclohexenol **23** (Scheme 4) with lipase PS^35^ gave the corresponding (*R*)-acetate **24** in 40% yield and 99% *ee*, along with 53% of the remaining alcohol in 95% *ee*. Derivatisation of the alcohol (*R*)-**23** to an α-bromoacetal followed by a highly diastereoselective radical cyclisation afforded the building block (1*R*,2*S*)-**26**, which was further elaborated into the spirocyclic alkaloids (+)-nitramine (**27**) and (+)-isonitramine (**28**). Similarly, its enantiomer (1*S*,2*R*)-**26** was transformed into (–)-sibirine.^40^

**Scheme 5:**
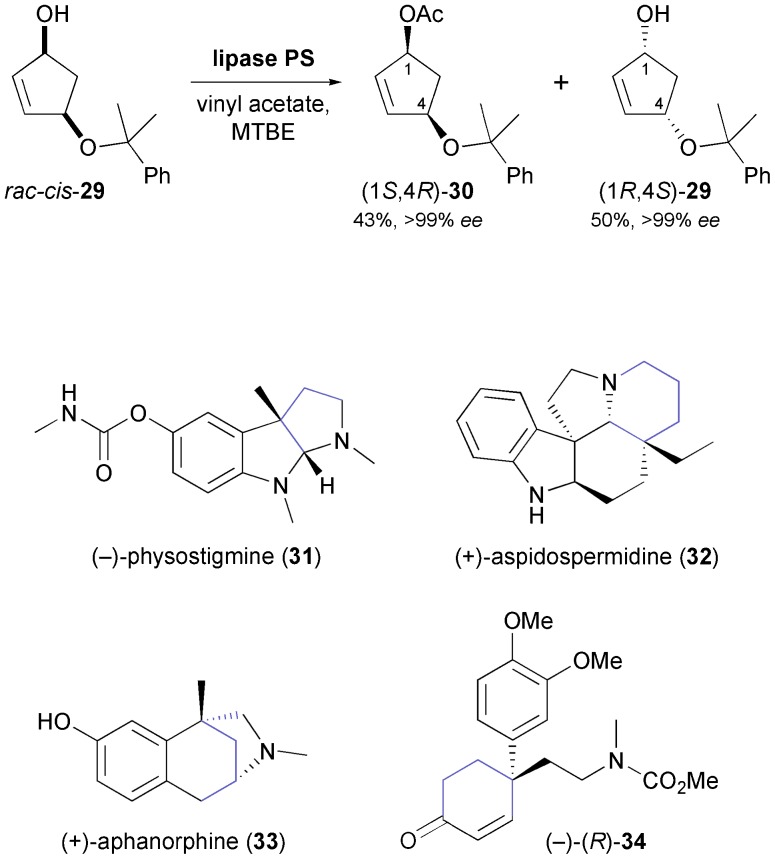
Lipase-catalysed kinetic resolution of building block **29**, and examples of alkaloids prepared from this building block.

Ogasawara and co-workers have developed a chemo-enzymatic route towards several cyclopentanoid chiral building blocks, using cyclopentadiene as starting material.^41^ The asymmetric key step is the kinetic resolution of *rac-cis*-**29** employing lipase PS^35^ in methyl *tert*-butyl ether (MTBE) at room temperature (Scheme 5). This transformation proceeds with excellent enantioselectivity, affording both the (–)-(1*S*,4*R*)-acetate **30** and the (+)-(1*R*,4*S*)-alcohol **29** in optically pure form (*ee* > 99%) and in high isolated yields (43% and 50%, respectively). Compound **29** has served as starting material in the (formal) total synthesis of various indole alkaloids, including (–)-physostigmine (**31**) and (+)-aspidospermidine (**32**), as well as the benzomorphan alkaloid (–)-aphanorphine (**33**, Scheme 5).^42^ In addition, it has been elaborated into the cyclohexenone derivative **34**, which is an intermediate *en route* to *Sceletium* alkaloids.^43^

**Scheme 6:**
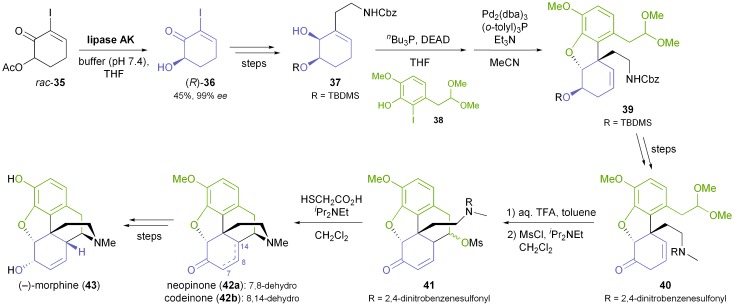
Fukuyama's chemo-enzymatic total synthesis of (–)-morphine (**43**).

While the chiral building blocks discussed so far have been used in multiple syntheses of alkaloids, lipase catalysis has also been employed to access some ‘purpose-made’ chiral intermediates for single synthetic endeavours. A notable example is Fukuyama's total synthesis of (–)-morphine (**43**, Scheme 6), which uses the alcohol **36**, obtained in 99% *ee* by hydrolytic kinetic resolution of the corresponding acetate **35** using lipase AK.^35,44^ All five stereocentres of the target molecule were established from **36** with excellent diastereocontrol, and (–)-morphine was obtained in 5% overall yield over 17 linear steps.

#### 2.1.2 Piperidine building blocks

**Scheme 7:**
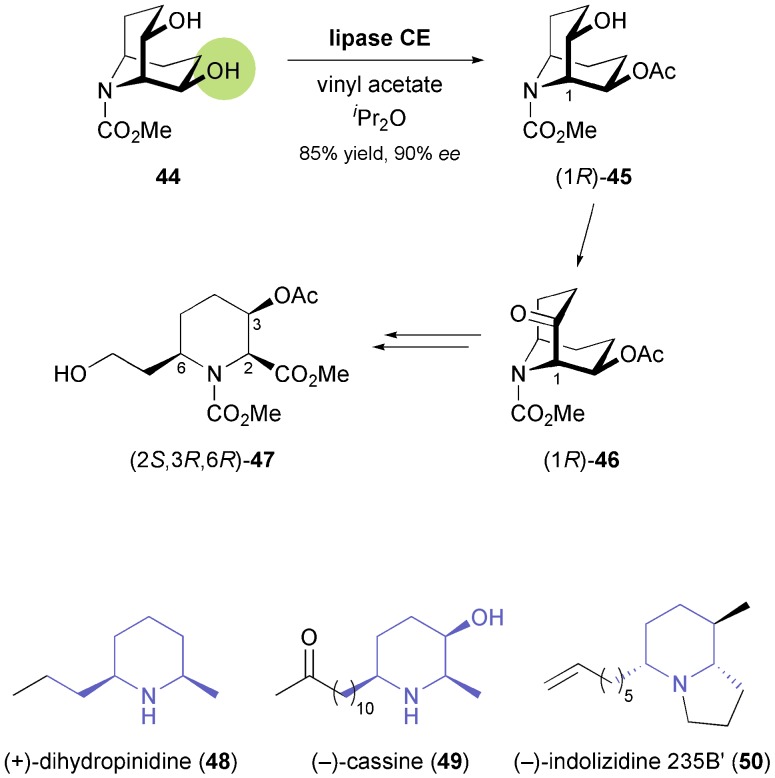
Lipase-catalysed desymmetrisation of diol **44**, its conversion into the trisubstituted piperidine derivative **47**, and examples of alkaloids prepared from this building block.

The first nitrogen-containing chiral building block derived from an enzymatic reaction was introduced to the asymmetric synthesis of alkaloids in 1992: Momose and co-workers have investigated the stereoselective transesterification of *N*-protected *meso*-9-azabicyclo[3.3.1]nonanediol **44** (Scheme 7), as well as the stereoselective hydrolysis of the corresponding diacetate, catalysed by various lipases.^45^ In both cases, a commercial lipase from *Humicola lanuginosa* ^35^ gave the best results, providing access to both enantiomers of the monoacetate **45** in high yield and good enantiomeric excess (*ee* = 90% for *R*, 80% for *S*). The monoacetate was oxidised to the corresponding ketone **46**, which could be obtained in optically pure form by a single recrystallisation from diisopropyl ether. This ketone, in turn, was transformed into the trisubstituted piperidine building block **47** *via* enol ether formation and ozonolytic cleavage of the bicyclic core structure (Scheme 7). Compound **47** proved useful for further elaboration into different piperidine alkaloids, such as (+)-dihydropinidine (**48**) and (–)-cassine (**49**),^45,46^ as well as indolizidines and quinolizidines sequestered by the poison-dart frogs of the *Dendrobatidae* family, *e.g.* (–)-indolizidine 235B′ (**50**, Scheme 7).^47^

**Scheme 8:**
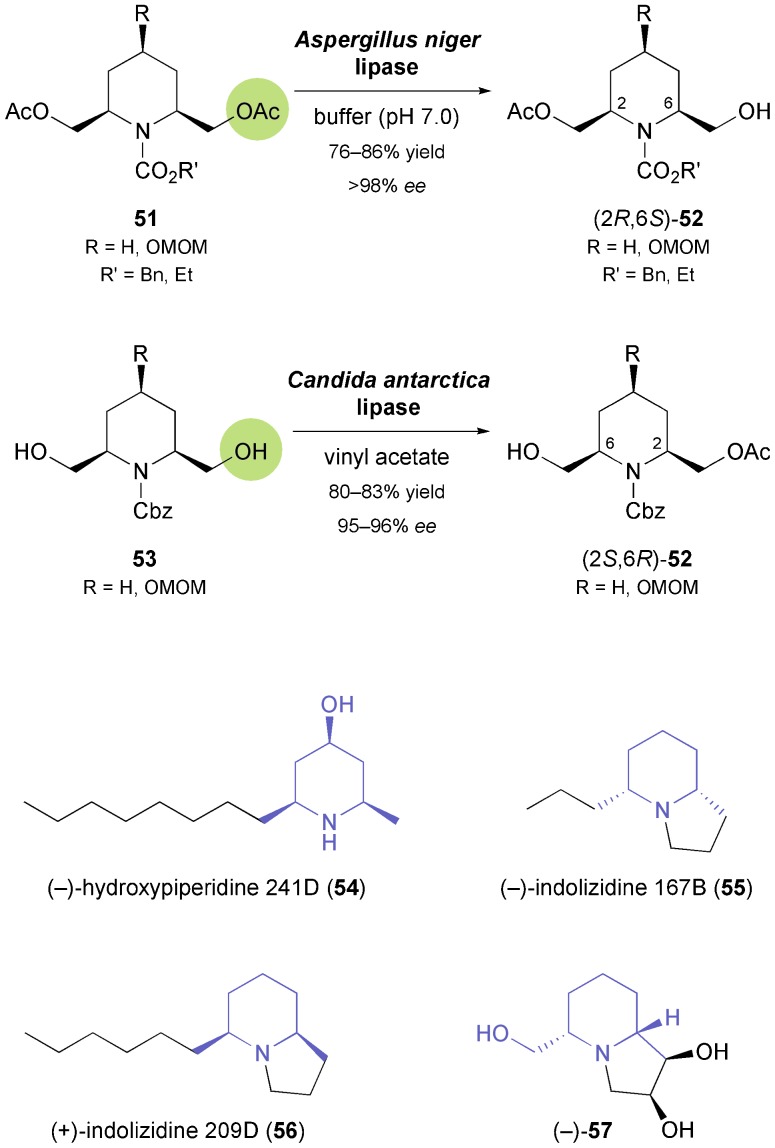
Biocatalytic preparation of both enantiomers of the 2,6-disubstituted piperidine building block **52**, and examples of alkaloids prepared from this building block.

The obvious advantages of nitrogen-containing chiral building blocks in the asymmetric synthesis of alkaloids led several other research groups to investigate biocatalytic methods for their preparation, and piperidine derivatives have attracted particular interest. Chênevert and Dickman have investigated the hydrolytic desymmetrisation of *meso*-diacetates **51** (Scheme 8) with various lipases and have identified the enzyme from *Aspergillus niger* as the most selective biocatalyst.^48^ The (2*R*,6*S*)-monoacetates **52** were isolated in good yields (76–92%) and excellent optical purity (*ee* > 98%), but the reaction took several days (72–108 h) to complete. Still, the authors could demonstrate the synthetic utility of the method by converting the monoacetates into (+)-dihydropinidine (**48**, Scheme 7) and the dendrobate frog alkaloids (+)-hydroxypiperidine 241D (**54**) and (–)-indolizidine 167B (**55**, Scheme 8).^48b,49^ Later studies avoided the time-consuming hydrolysis protocol and focused on the preparation of the opposite enantiomer of **52** by lipase-catalysed stereoselective monoacetylation. The lipases from *Candida antarctica* and *Candida cylindracea* (the latter employed in the ionic liquid BMIM-PF_6_ as reaction medium) were found to be suitable enzymes for this transformation, affording (2*S*,6*R*)-**38** in 80% yield (*ee* = 95%) and 90% yield (*ee* = 98%), respectively.^50^ In the former case, the piperidine intermediate was further transformed into the non-natural enantiomer of the *Dendrobatidae* alkaloid indolizidine 209D [(+)-**56**],^50*a*^ while in the latter it was elaborated into polyhydroxylated indolizidines and quinolizidines, *e.g.* (–)-**57** (Scheme 8), *via* a sequence featuring ring-closing metathesis and OsO_4_-catalysed dihydroxylation as key steps.^50*b*^

**Scheme 9:**
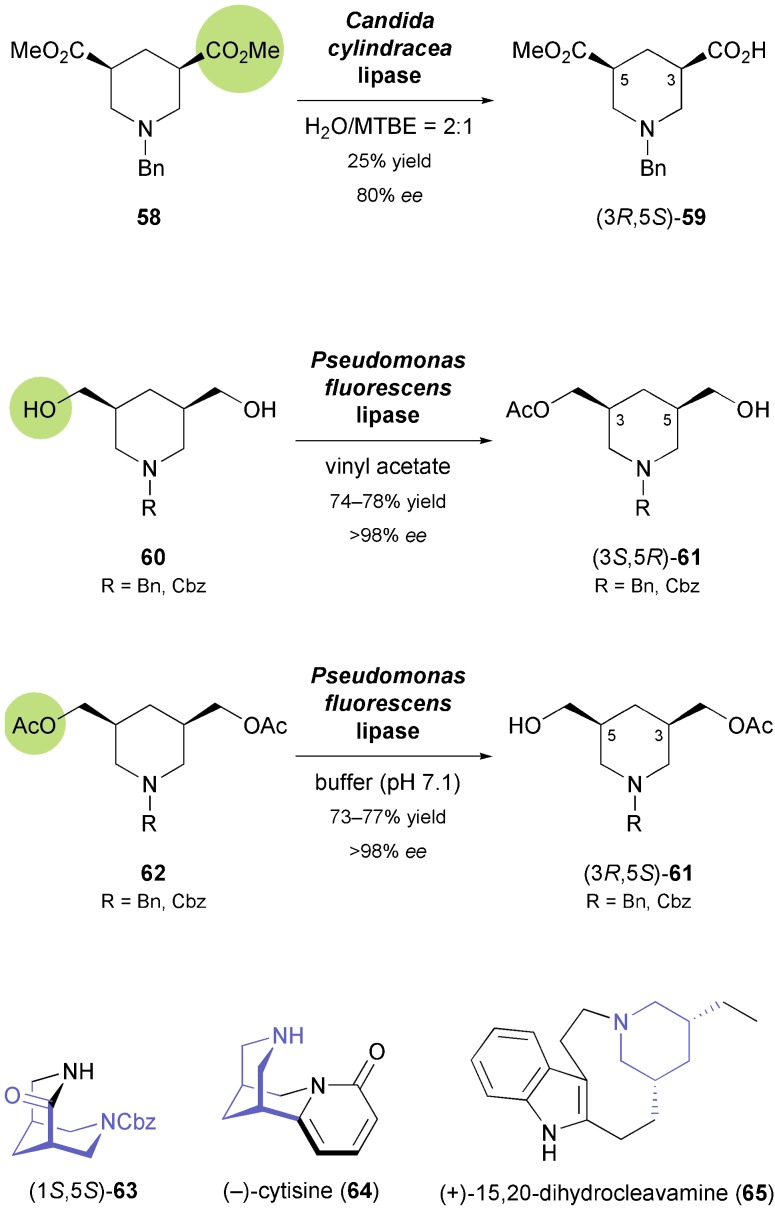
Biocatalytic preparation of the 3,5-disubstituted piperidine building blocks **59** and **61**, and examples of alkaloids prepared from **61**.

3,5-*cis*-Disubstituted piperidines also form a common structural motif in natural products, for example in indole alkaloids of the ibogan and tacaman type, or in (–)-sparteine, which has found broad application as a chiral ligand. Lesma and co-workers first tried to establish an asymmetric entry to 3,5-disubstituted piperidine building blocks *via* stereoselective enzymatic hydrolysis of *meso*-diesters **58** (Scheme 9), but both yield and optical purity of the obtained monoesters **59** were only moderate.^51^ Stereoselective monoacetylation of the analogous piperidine-3,5-dimethanols **60** (Scheme 9) catalysed by the lipase from *Pseudomonas fluorescens* turned out to be more efficient, providing the (3*S*,5*R*)-enantiomer of monoacetates **61** in good yield (74–78%) and excellent optical purity (*ee* > 98%).^52^ The opposite enantiomer could conveniently be accessed by hydrolytic desymmetrisation of the diacetates **62** using the same enzyme. The chiral building blocks thus obtained served as a basis for the preparation of several compounds featuring *cis*-fused piperidine rings as core structure, *e.g.* the 3,7-diazabicyclo[3.3.1]nonane derivative **63**,^53^ the *Leguminosae* alkaloid (–)-cytisine (**64**, Scheme 9),^54^ and a truncated (+)-sparteine analogue.^55^ Furthermore, the conversion of derivatives of **61** into the ibogan type indole alkaloid (+)-dihydrocleavamine (**65**), featuring an unusual tetracyclic framework that contains a 9-membered ring, has been reported.^56^

**Fig. 2:**
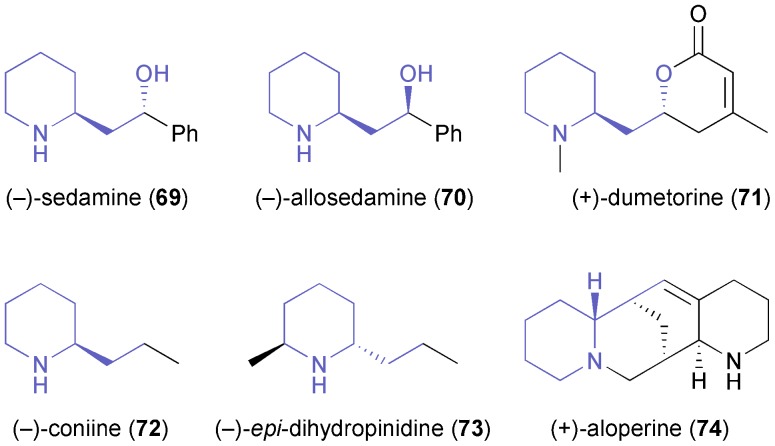
Examples of alkaloids prepared from building block **67**.

**Scheme 10:**
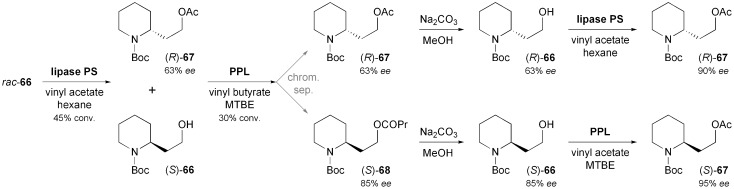
Preparation of both enantiomers of the 2-substituted piperidine building block **67** *via* a triple kinetic resolution protocol employing enantiocomplementary lipases.

In comparison to the di- or polysubstituted piperidines described above, monosubstituted derivatives have attracted less attention, although several monosubstituted piperidine alkaloids are known. Passarella, Riva, and co-workers have investigated *N*-Boc-piperidine-2-ethanol **66** as chiral building block, but this compound proved a challenging substrate for lipase-catalysed kinetic resolution due to its conformational flexibility and the distance of the alcohol moiety from the stereogenic centre.^57^ The enantioselectivities observed were generally low (*E* ≤ 8), but the identification of two enantiocomplementary enzymes–(*S*)-selective porcine pancreatic lipase (PPL), and (*R*)-selective lipase PS (from *Burkholderia cepacia*)^35^–allowed the preparation of both enantiomers of acetate **67** in decent optical purity (*ee* = 90% for *R*, 95% for *S*) *via* a complex, but scalable triple kinetic resolution protocol (Scheme 10). The optically enriched building blocks were transformed into both enantiomers of sedamine (**69**) and allosedamine (**70**, Fig. 2) in three additional steps. In a similar fashion, (+)-dumetorine (**71**),^58^ (–)-coniine (**72**),^59^ and (–)-*epi*-dihydropinidine (**73**) were prepared, whereby in the latter case the *N*-Boc group served as a lithiation director in the introduction of the 6-methyl substituent.^58^ Furthermore, the lupinine alkaloid (+)-aloperine (**74**) was synthesised from (*R*)-**67** in a 12-step sequence that featured the use of a laccase–TEMPO system for the oxidation of primary alcohol moieties, and of a Diels–Alder reaction for setting up the correct stereochemistry at three chiral centres.^60^

#### 2.1.3 Nitrone building blocks

**Scheme 11:**
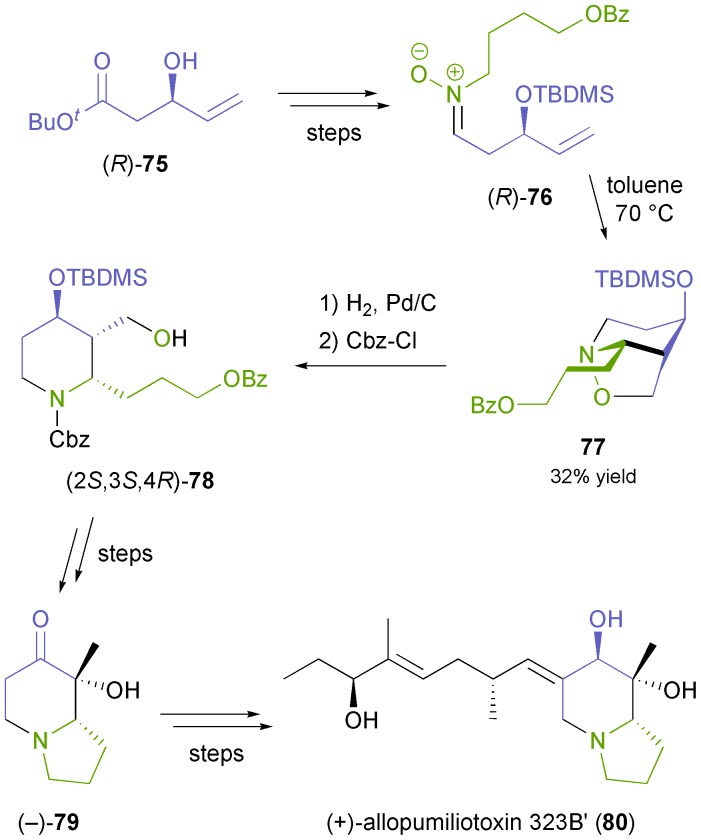
Synthesis of (+)-allopumiliotoxin 323B′ (**80**) from the lipase-derived building block (*R*)-**75**.

Optically active nitrones represent chiral building blocks that are particularly interesting for the synthesis of nitrogen heterocycles. The nitrone moiety can react with olefins in inter- or intramolecular (3 + 2)-cycloadditions, which often proceed with excellent regio- and diastereoselectivity, and furthermore this functional group can provide a nitrogen atom present in the target molecule. These features were exploited by Holmes and co-workers,^61^ who prepared nitrone **76** from the chiral alcohol (*R*)-**75** (Scheme 11), which was obtained in 47% yield and 92% *ee* by kinetic resolution of the racemate using lipase PS^35^ under previously reported conditions.^62^ Intramolecular cyclisation of **76** afforded a separable mixture of isomers, of which the major one (**77**, 32%) was converted into the indolizidine (–)-**79**,^61*a*^ and further into the dendrobate frog alkaloid (+)-allopumiliotoxin 323B′ (**80**).^61*b*^

**Scheme 12:**
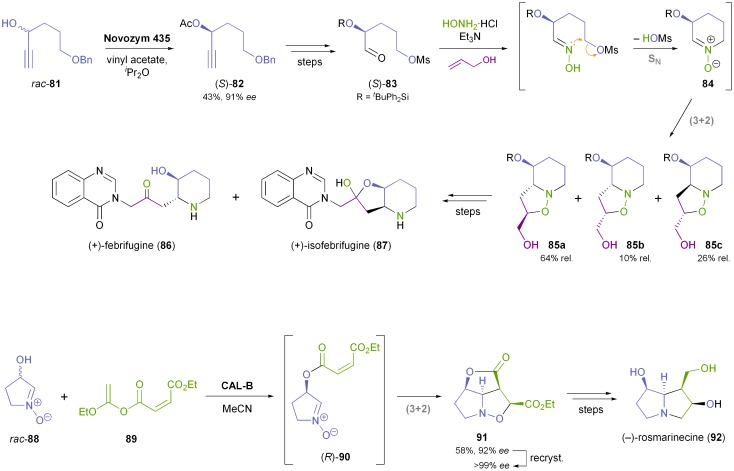
Cascade reactions involving nitrone–olefin (3 + 2)-cycloadditions in the chemo-enzymatic syntheses of (+)-febrifugine (**86**), (+)-isofebrifugine (**87**), and (–)-rosmarinecine (**92**).

Hatakeyama and co-workers have devised a synthetic sequence towards the antimalarial *Hydrangea* alkaloids (+)-febrifugine (**86**) and (+)-isofebrifugine (**87**), in which the nitrone **84** is a key intermediate (Scheme 12).^63^ Kinetic resolution of alcohol **81** using immobilised *Candida antarctica* lipase B (CAL-B; Novozym 435)^35^ provided the corresponding (*S*)-acetate **82** in 43% yield and 91% *ee* (enantioselectivity *E* = 50). The key step of the synthesis is a cascade nitrone formation–cycloaddition reaction, which joins three molecules and sets up two rings and two chiral centres in one operation, albeit in only moderate diastereoselectivity. The mixture of isomers obtained was transformed into the target alkaloids **86** and **87** in six additional steps. An even more elegant one-pot procedure forms the cornerstone of Kita's asymmetric synthesis of (–)-rosmarinecine (**92**): the racemic hydroxynitrone **88** was stereoselectively acylated with the maleate ester **89** using immobilised CAL-B (Chirazyme L-2),^35^ and the acylation product underwent a spontaneous intramolecular (3 + 2)-cycloaddition to afford compound **91**, featuring 4 contiguous stereocentres, as a single diastereomer in 58% yield and 92% *ee* (Scheme 12). Recrystallisation and minor functional group interconversions completed the synthesis of optically pure **92**, which was obtained in 45% overall yield from *rac*-**88**.^64^ An improved procedure for the preparation of *rac*-**88** published some years later even rendered the use of protective groups unnecessary.^65^

### 2.2 Toluene Dioxygenase

**Scheme 13:**
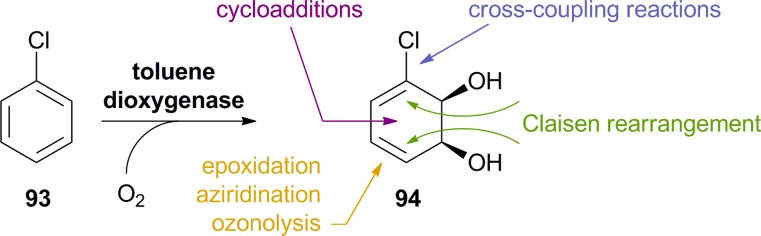
Preparation of aromatic *cis*-dihydrodiols, *e.g.* **94**, by biocatalytic dioxygenation of arenes, and some general methods for their further functionalisation.

Arene *cis*-dihydrodiols are versatile chiral building blocks offering ample possibilities for further functionalisation (Scheme 13).^66^ While the asymmetric preparation of these labile compounds represents an enormous challenge for chemical methods, they are formed with excellent selectivity from the corresponding arenes by the action of bacterial dioxygenases. Toluene dioxygenase (TDO) from *Pseudomonas putida* is the best-known of these enzymes, and the *cis*-dihydroxylation of various simple arenes by *Pseudomonas* has been reported by David Gibson and co-workers as early as 1968.^67^ It took almost two decades until the preparative value of this biotransformation was recognised, but since the late 1980s, biocatalytically prepared arene *cis*-dihydrodiols have been used in the asymmetric synthesis of numerous natural products, including many alkaloids.^66^ Early studies relied on *Pseudomonas putida* 39/D, a mutant strain developed by Gibson's group, for fermentative production of the desired metabolites, but later the responsible toluene dioxygenase has been cloned and heterologously expressed in *E. coli*, which soon became the preferred biocatalyst. Today, several optically pure arene *cis*-dihydrodiols (obtained by TDO catalysis) are commercially available.

#### 2.2.1 TDO in the synthesis of pyrrolizidine and iminocyclitol alkaloids

**Scheme 14:**
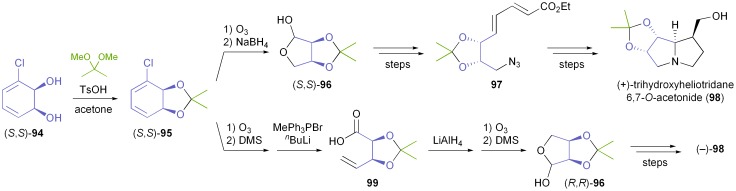
Stereodivergent synthesis of trihydroxyheliotridane acetonide (**98**) from the TDO-derived diol **94**.

The first asymmetric synthesis of an alkaloid from an arene *cis*-dihydrodiol was reported by Hudlicky *et al.* in 1990:^68^ The authors prepared both enantiomers of acetonide-protected trihydroxyheliotridane **98**, in nine steps for the (+)-isomer and twelve for its (–)-counterpart, from chlorobenzene **93**. Thereby, they made elegant use of ozonolysis and selective redox transformations to obtain either enantiomer of the key intermediate **96** from the same *cis*-dihydrodiol (Scheme 14).

**Fig. 3:**
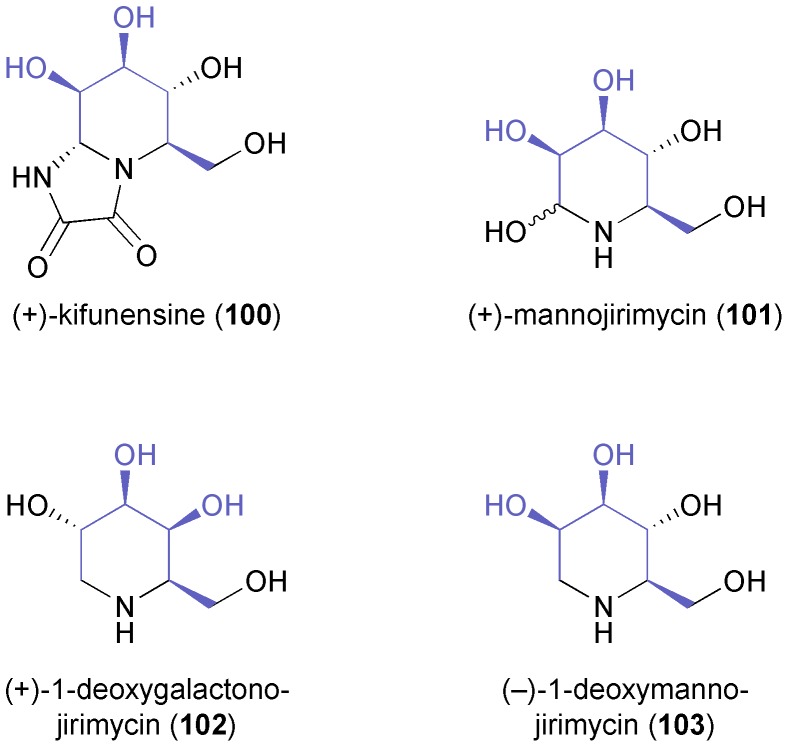
Examples of iminocyclitol alkaloids prepared from TDO-derived arene *cis*-dihydrodiols.

A related ozonolysis approach (in this case cleaving only one double bond) has been followed in the synthesis of the iminocyclitols (+)-kifunensine (**100**), (+)-mannojirimycin (**101**), (+)-1-deoxygalactonojirimycin (**102**), and (–)-1-deoxymanno-jirimycin (**103**, Fig. 3), which are glycosidase inhibitors with potential anti-viral properties.^69^

#### 2.2.2 TDO in the synthesis of *Amaryllidaceae* isocarbostyril alkaloids

**Fig. 4:**
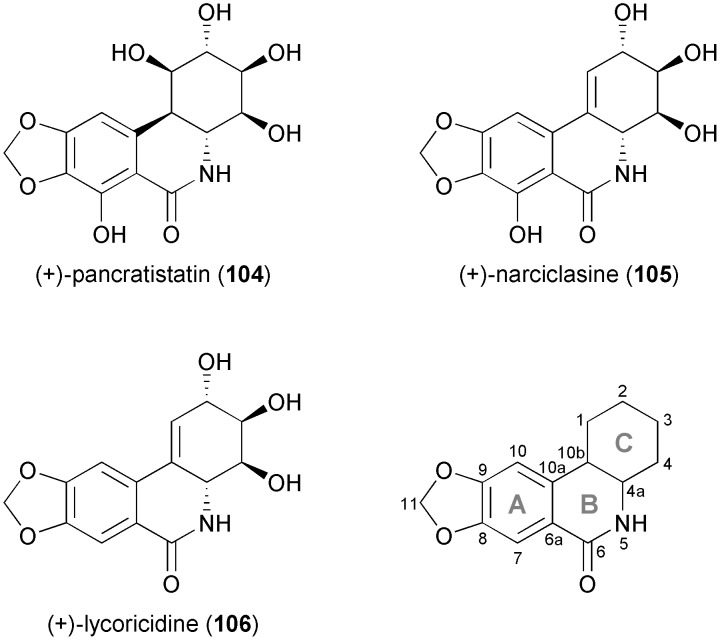
Structures of pancratistatin (**104**), narciclasine (**105**) and lycoricidine (**106**), and general ring denotation and atom numbering conventions for isocarbostyril alkaloids.

Over the last 20 years, Hudlicky's research group has made extensive use of arene *cis*-dihydrodiols as building blocks in natural product synthesis, most notably in the preparation of the cytotoxic *Amaryllidaceae* alkaloids pancratistatin (**104**), narciclasine (**105**), lycoricidine (**106**), and their analogues (Fig. 4), which exhibit potent *in vitro* and *in vivo* anti-cancer activity.^66,70^

**Scheme 15:**
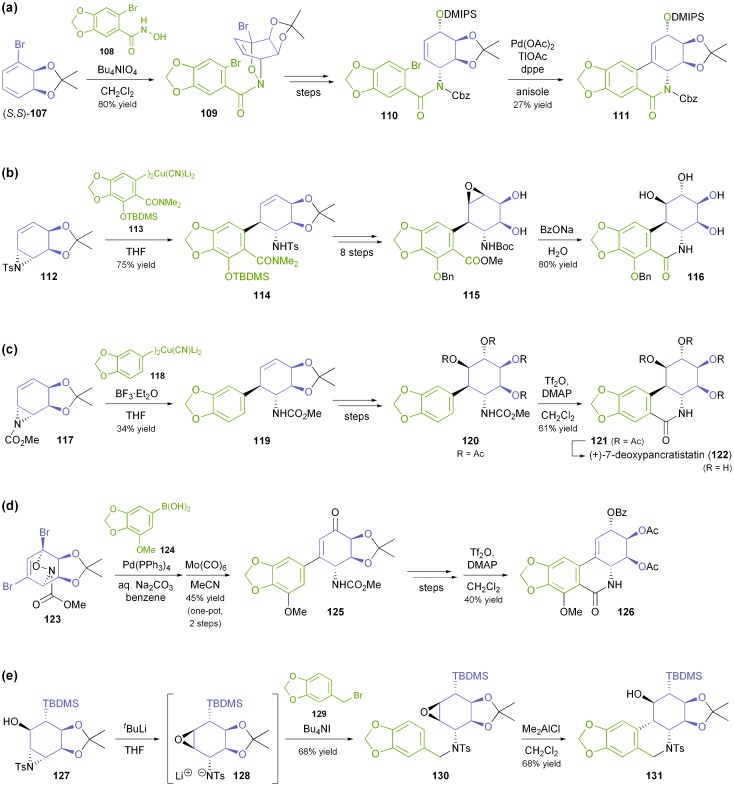
Different strategies for construction of the B ring of isocarbostyril alkaloids: (a) nitroso-Diels–Alder reaction and Heck reaction, (b) aziridine ring-opening and intramolecular ester aminolysis, (c) aziridine ring-opening and Bischler–Napieralski cyclisation, (d) Suzuki cross-coupling and Bischler–Napieralski cyclisation, and (e) aza-Payne rearrangement/alkylation and intramolecular epoxide ring-opening.

In all these synthetic studies, the biocatalytically prepared *cis*-diol served as precursor for the C-ring of the target compounds, while different strategies were explored for the construction of ring B: (*i*) In the case of (+)-lycoricidine (**106**), the first *Amaryllidaceae* alkaloid obtained by this chemo-enzymatic approach, the piperonyl unit **108** was attached to the diol building block **107** *via* an acyl-nitroso–diene [4 + 2] cycloaddition (nitroso-Diels–Alder reaction).^71^ Subsequent closure of the B-ring by a Heck reaction proved problematic, as desilylation and transacetylation occurred as side reactions, resulting in a yield of only 27% (Scheme 15a). (*ii*) Later studies focused on nucleophilic opening of aziridines derived from the arene *cis*-diol as a way to connect rings A and C, and this approach was first used in the asymmetric synthesis of (+)-pancratistatin (**104**):^72^ The tosylaziridine **112**, obtained in 4 steps from bromobenzene, was coupled with the cyanocuprate **113**, whose dimethylamide moiety was meant to serve as direct precursor of the amide functionality in the target compound. However, the intended intramolecular transamidation on intermediate **114** could not be achieved, making various additional functional group interconversions necessary (Scheme 15b). (*iii*) This problem was addressed in the synthesis of 7-deoxypancratistatin (**122**, Scheme 15c), where the tosylaziridine was replaced by the corresponding carbomethoxy analogue **117**, and cyanocuprate **118**–lacking the amide moiety of **113**–was used as electrophile.^73^ Ring-closure was achieved in fair yield (61%) by a Bischler–Napieralski-type cyclisation. (*iv*) The same method for closing ring B was employed in the synthesis of narciclasine (**105**, Scheme 15d), while the strategy for coupling the diol and piperonyl units was again changed:^74^ The presence of an additional bromine atom in the acetonide **123** made Suzuki coupling possible, avoiding the need for the rather unstable cyanocuprate. For the construction of **123**, the authors returned to a nitroso-Diels–Alder approach, providing an elegant means of differentiating between the two vinylic bromide moieties of the TDO-derived *cis*-diol. (*v*) In the synthesis of 10b-*epi*-7-deoxypancratistatin the B ring was constructed *via* an aza-Payne rearrangement on aziridine **127** and Lewis-acid catalysed intramolecular epoxide ring-opening on the resulting tosylamide **130** (Scheme 15e).^74b,75^ Several derivatives of the natural *Amaryllidaceae* alkaloids have been prepared following variations of these synthetic strategies, including a carboline analogue,^76^ derivatives featuring various functional groups at C1,^77^ and others.^74b,78^

**Scheme 16:**
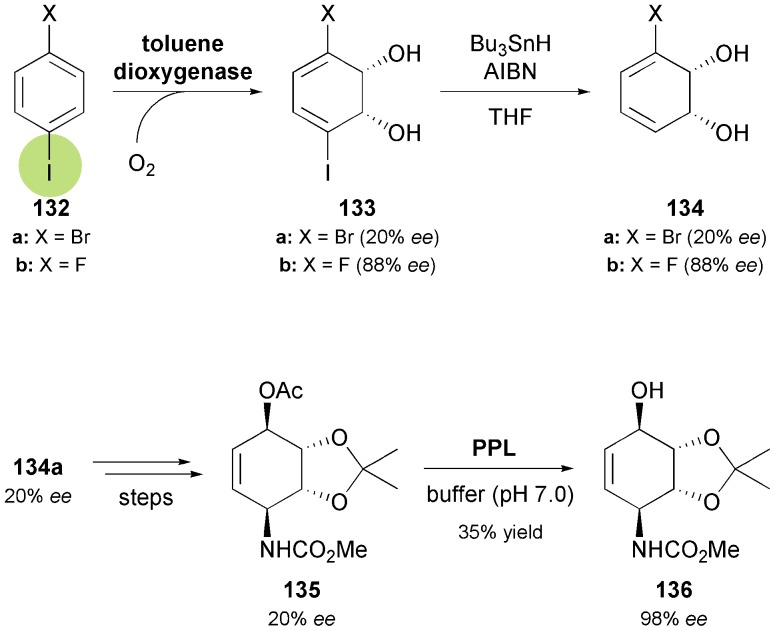
Chemo-enzymatic preparation of (*R*,*R*)-3-halocyclohexadiene-1,2-diols **134** *via* TDO-catalysed oxygenation of *para*-dihalobenzenes **132**, and lipase-catalysed kinetic resolution of conduramine A derivative **135**.

One of the challenges when using arene *cis*-dihydrodiols as building blocks in asymmetric synthesis arises from the fact that the highly stereoselective TDO-catalysed dioxygenation only provides access to one enantiomer of the desired metabolite. As a consequence, enantiodivergent syntheses are much more difficult to realise by this approach than by hydrolase catalysis, where stereocomplementary enzymes are often available and reactions can be run in two ‘modes’ (acylation *vs.* hydrolysis). In the particular case of the *Amaryllidaceae* alkaloids, two strategies to overcome this problem, and thereby make the non-natural enantiomers available, have been developed. Hudlicky's research group has carried out the TDO-catalysed oxidation of *para*-dihalobenzenes **132a** and **132b** ^79^ followed by reductive removal of the iodine atom, to obtain the (*R*,*R*)-diols **134a** and **134b** in 20% and 88% enantiomeric excess, respectively (Scheme 16).^74b,80^ In the first case, the optically enriched intermediate was converted into the conduramine A derivative **135**, which was subjected to lipase-catalysed kinetic resolution, affording the alcohol **136** in 98% *ee* (Scheme 16). From this building block, (–)-7-deoxypancratistatin (*ent*-**121**) was synthesised in eight steps following the approach developed for the preparation of the natural alkaloid. The fluorodiol **134b**, on the other hand, was not easily converted into **136**, but instead gave the corresponding ketone, which can also serve as building block for alkaloid synthesis.

**Scheme 17:**
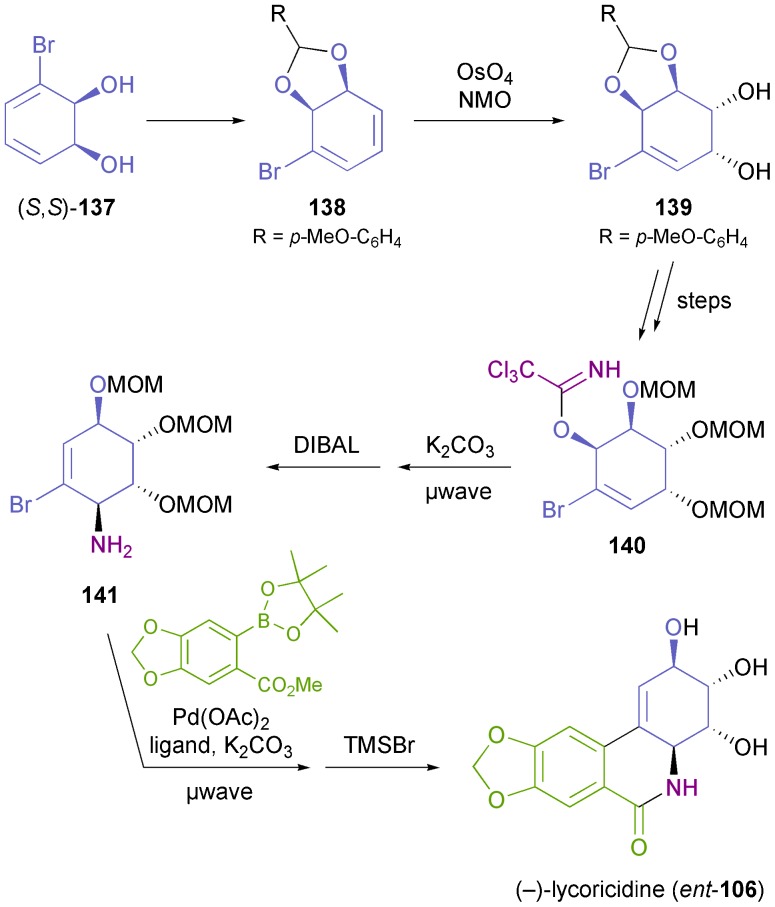
Chemo-enzymatic synthesis of (–)-lycoricidine (*ent*-**106**) from TDO-derived diol (*S*,*S*)-**137**.

A completely different approach towards the *ent*-series of *Amaryllidaceae* alkaloids has been presented by Banwell and co-workers, who prepared non-natural (–)-lycoricidine (*ent*-**106**) from bromodiol (*S*,*S*)-**137**, the same compound used by Hudlicky for the preparation of the (+)-enantiomer.^81^ For the *ent*-series, the *cis*-vicinal diol moiety of the target compound can obviously not derive directly from the hydroxyl groups in **137**. Instead, a diol of correct absolute configuration was established by an OsO_4_-catalysed dihydroxylation reaction on derivative **138**. Further key transformations of the 10-step synthesis are an Overman rearrangement providing the amine moiety in **141**, and a Suzuki coupling used to establish the B ring (Scheme 17). The syntheses of 3-*epi-ent*-lycoricidine, 4-deoxy-3-*epi-ent*-lycoricidine, and (–)-narciclasine (*ent*-**104**) following similar lines have also been reported.^81,82^

#### 2.2.3 TDO in the synthesis of morphinan alkaloids

**Fig. 5:**
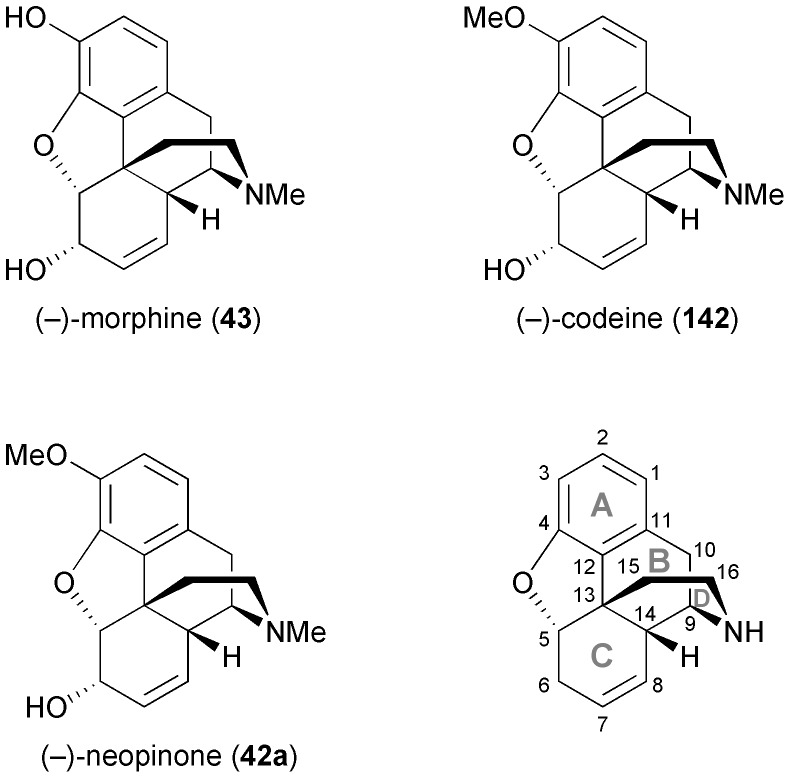
Structures of morphine (**43**), codeine (**142**) and neopinone (**42a**), and general ring denotation and atom numbering conventions for morphinan alkaloids.

**Scheme 18:**
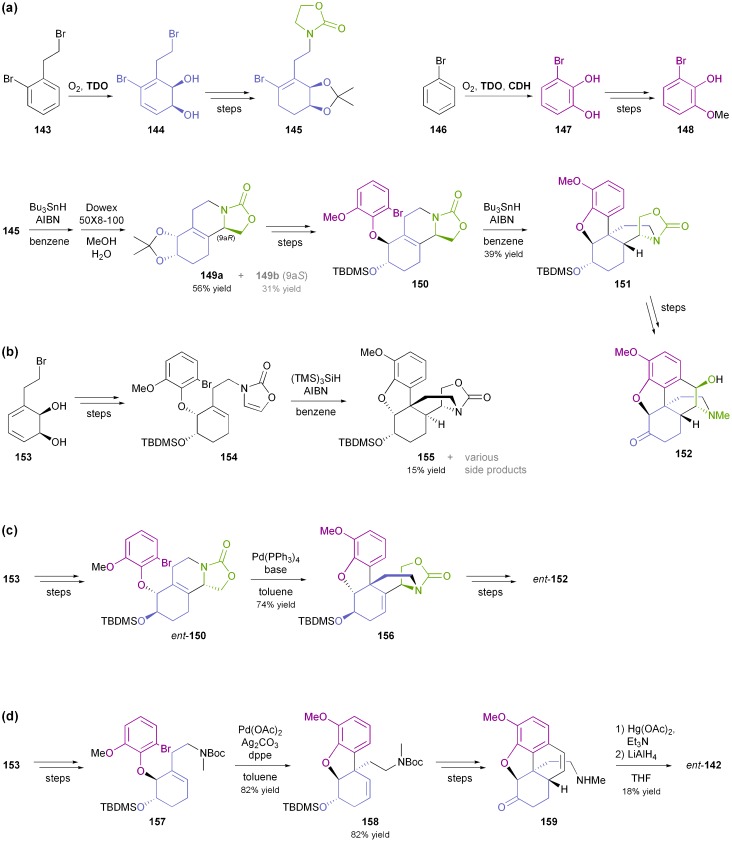
Chemo-enzymatic synthesis of morphinan alkaloids: (a) preparation of building blocks **145** and **148** using biocatalytic dioxygenation, and synthesis of morphinan **152** from these building blocks using radical cyclisations, (b) radical cyclisation cascade affording compound **155**, (c) synthesis of *ent*-**152** from TDO-derived diol **153** by a Heck-cyclisation approach, and (d) synthesis of (+)-neopinone (*ent*-**142**) from TDO-derived diol **153** *via* Heck-cyclisation and hydroamination as key steps.

Considerable effort has also been devoted to the synthesis of morphinan alkaloids (Fig. 5) from arene *cis*-dihydrodiols.^70a,83^ Hudlicky's research group has first established a route to the complete morphinan core structure from two building blocks obtained with the help of biocatalytic dioxygenation:^84^ The oxidation of **143** by TDO afforded the diol **144**, as the regio- and stereochemistry of the bioconversion is directed by the larger bromoethyl substituent. The diol was subsequently converted into compound **145**. Furthermore, the incubation of bromobenzene **146** with an *E. coli* strain expressing both TDO and catechol dehydrogenase (CDH) yielded bromocatechol **147**, which was transformed into the arene building block **148**. A radical cyclisation strategy was then followed to arrive at a key intermediate **151**, and finally the morphinan skeleton was established (in non-natural absolute configuration) by C10–C11 ring closure (Scheme 18a). Studies aimed at preparing the natural enantiomer from building block **153** using a related cascade radical cyclisation of intermediate **154** as key step met with limited success, as the cyclisation proceeded with low diastereoselectivity and afforded the *epi*-C14 isomer **155** as the main product (Scheme 18b).^84*b*^ An alternative route based on an intramolecular Diels–Alder reaction was also explored but did not give satisfactory results either.^85^ An entry to the natural series of morphinan alkaloids was finally established by a Heck cyclisation approach (Scheme 18c), using the chemo-enzymatically prepared building blocks **148** and **153**.^86^ A Heck-based enantiodivergent synthesis of both enantiomers of codeine (**142**), which uses mercury(ii)-catalysed hydroamination for closing ring D (Scheme 18d), has also been reported recently.^87^ A further variation of this route has been employed in the synthesis of *ent*-neopinone (*ent*-**42a**), in which the D ring is closed by a 1,6-conjugate addition of the amine to C9.^88^

#### 2.2.4 Recent application of TDO in the synthesis of various plant alkaloids

**Fig. 6:**
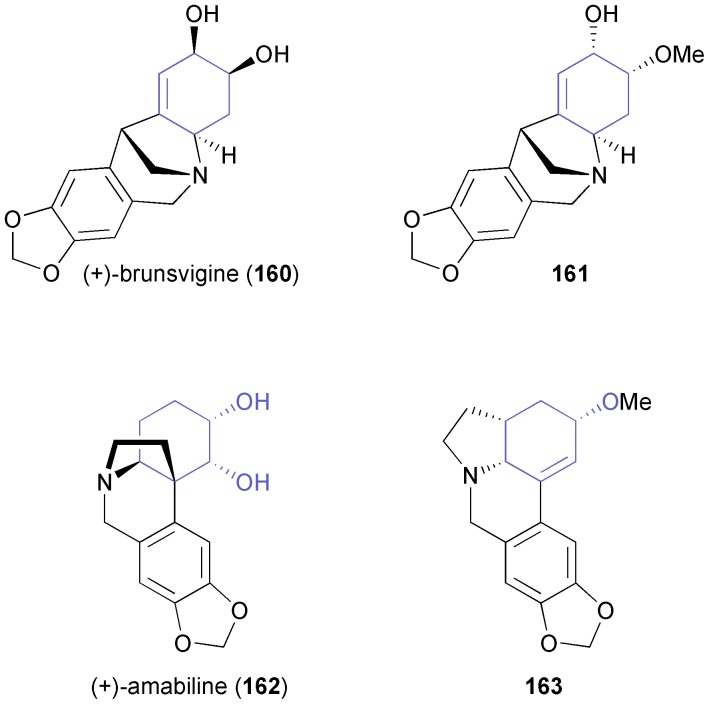
Examples of *Amaryllidaceae* alkaloids synthesised from TDO-derived building blocks.

In recent years, Banwell and co-workers have considerably broadened the application of arene *cis*-dihydrodiols in the synthesis of alkaloids.^89^ For instance, the authors have established a general route towards the montanine class of *Amaryllidaceae* alkaloids, which uses (*S*,*S*)-**94** (Scheme 14) as starting material and employs a radical cyclisation as well as a Pictet–Spengler reaction for construction of the pentacyclic target compounds. Thus, the closely related alkaloids (+)-brunsvigine (**160**) and (+)-nangustine (non-natural enantiomer in both cases), and the structure **161** preliminarily assigned to (+)-montabuphine (Fig. 6) have been prepared.^90^ The last synthesis demonstrated that montabuphine is not identical to structure **161**. Further synthetic studies on *Amaryllidaceae* alkaloids conducted by Banwell's group have resulted in the asymmetric preparation of (+)-amabiline (**162**) and the lycorine derivative **163** (Fig. 6) in 15 and 10 steps, respectively, from the TDO-derived building block (*S*,*S*)-**137** (Scheme 21).^91^

**Scheme 19:**
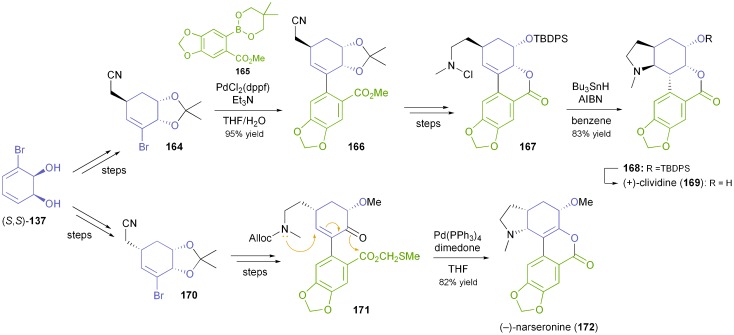
Synthesis of lycorenine-type alkaloids (+)-clividine (**169**) and (–)-narseronine (**172**) from TDO-derived diol **137**.

Banwell's group has also developed a chemo-enzymatic synthetic approach towards the lycorenine-type alkaloids (+)-clividine (**169**) and (–)-narseronine (**172**).^92^ Both syntheses employ Suzuki coupling as a key step and close ring D through the formation of the N–C11c bond, in the former case *via* a nitrogen-centred radical, in the latter by a conjugate addition/esterification cascade (Scheme 19). The structure assigned to the alkaloid nobilisitine A has been obtained in an analogous manner, and also in this case the spectral data indicated that the structural assignment was incorrect.^93^

### 2.3 Baker's yeast

**Scheme 20:**
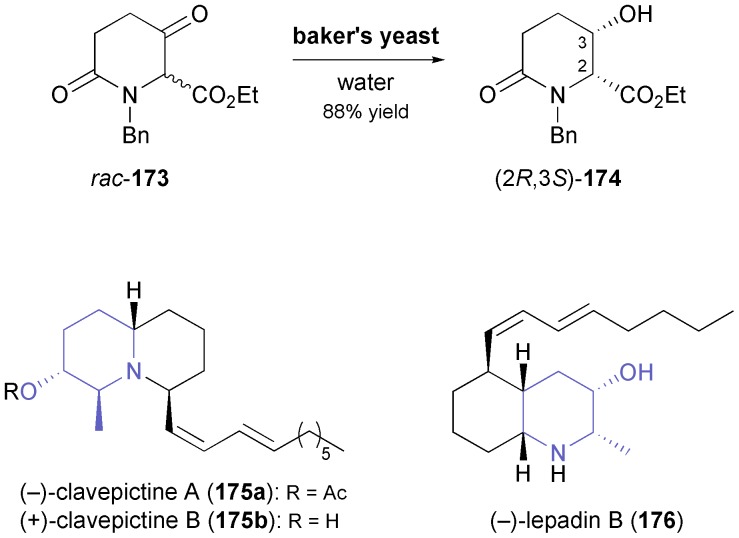
Asymmetric reduction/dynamic kinetic resolution of *rac*-**173** by bakers yeast affording building block **174**, and examples of alkaloids prepared from this building block.

While chiral alcohols are frequently used as building blocks in the synthesis of alkaloids, biocatalytic asymmetric reduction of the corresponding ketones has only rarely been explored as a way to access them.^94^ A notable exception is the highly functionalised piperidinol **174** (Scheme 20) described by Momose and co-workers:^95^ Although this compound can be obtained by kinetic resolution of the racemate using lipase PS^35^, the authors found it more efficient to subject the corresponding ketone **173** to reduction by baker's yeast. This biotransformation afforded (2*R*,3*S*)-**174** in 98% *ee* and 88% yield, as the chiral centre at C2 was dynamically resolved in the process. Several 2,6-disubstituted 3-piperidinols of all four possible diastereomeric forms have been prepared from **174** and used in the asymmetric synthesis of various alkaloids.^95,96^ For instance, the preparation of (–)-iso-6-cassine (*cf.* Scheme 7), clavepictines A [(–)-**175a**] and B [(+)-**175b**], and (–)-lepadin B (**176**) has been reported (Scheme 20).^97^

**Scheme 21:**
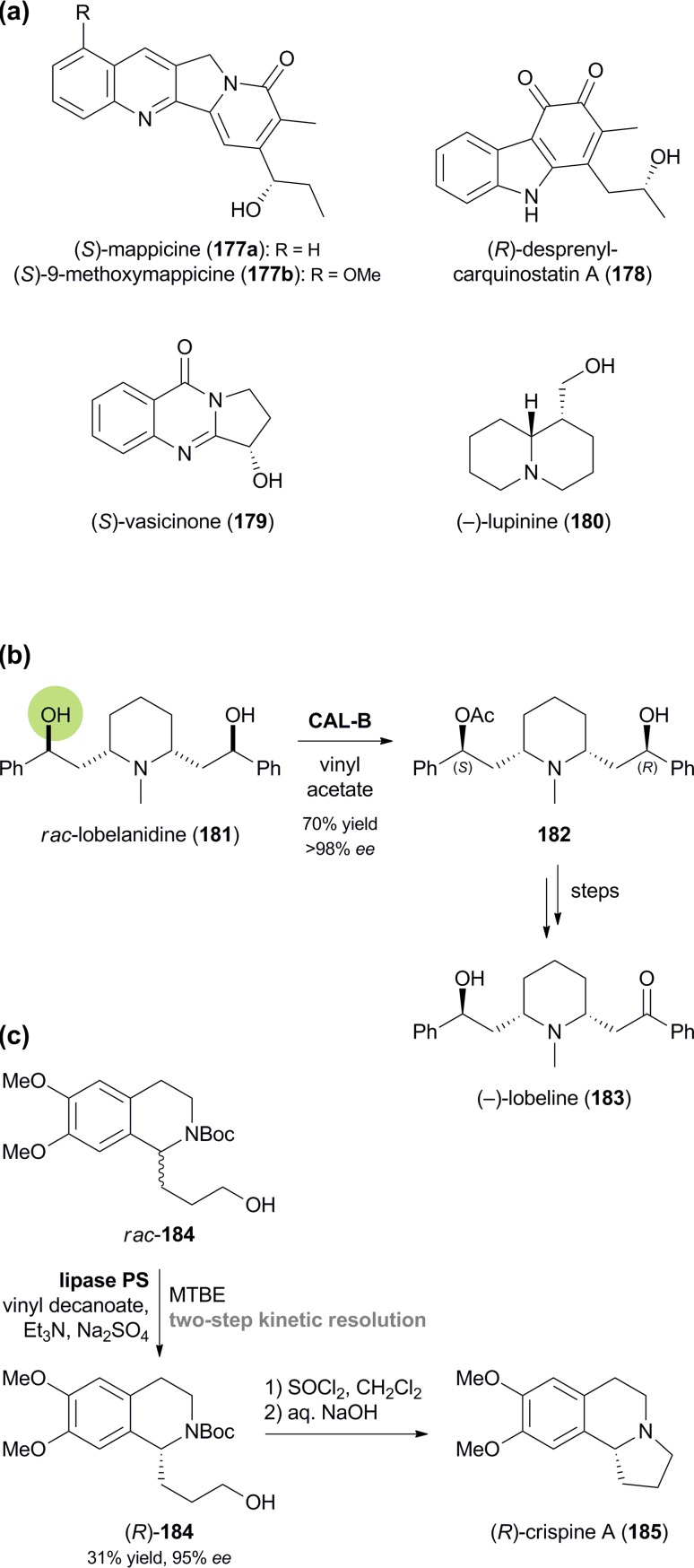
Lipase-catalysed kinetic resolution and desymmetrisation of alkaloids: (a) examples of alkaloids obtained by kinetic resolution, (b) desymmetrisation of lobelanidine (**181**) and conversion into (–)-lobeline (**183**), and (c) kinetic resolution of alcohol **184** and conversion into (*R*)-crispine A (**185**).

## 3. Biocatalytic kinetic resolution, desymmetrisation, and deracemisation of alkaloids

The enormous flexibility of the ‘chiral building block’ approach has enabled the chemo-enzymatic synthesis of alkaloids of remarkable structural complexity and diversity, often even in both enantiomeric forms. However, as chirality is introduced early in the synthetic sequence, an enantiodivergent synthesis requires several identical steps to be carried out independently for each enantiomer. In some cases it would clearly be more efficient to prepare the racemic alkaloid (or a late synthetic intermediate) and subject it to kinetic resolution. On the other hand, even if only one enantiomer of an alkaloid is to be synthesised, preparation of the racemate followed by deracemisation might be an attractive option. Biocatalysis offers possibilities for both these approaches; however, the sheer size and functional complexity of alkaloids make them challenging substrates even for enzymes, and hence examples are limited in number.

### 3.1 Lipase-catalysed kinetic resolution and desymmetrisation

Amongst the largest alkaloids that have been kinetically resolved are mappicine (**177a**) and 9-methoxymappicine (**177b**, Scheme 21a), two minor constituents of *Nothapodytes foetida*, which can be obtained in racemic form from the more abundant camptothecin. Kinetic resolution of the side-chain alcohol in **177a** and **177b** has been achieved by enantioselective hydrolysis of the corresponding acetates with baker's yeast or lipase PS^35^ (with enantioselectivities *E* ranging from 31 to >200), and by enantioselective acetylation with *Candida cylindracea* lipase (**177a**: *E* > 200, **177b**: *E* = 146).^98^ Similarly, a side-chain secondary alcohol has been resolved in the chemo-enzymatic preparation of the quinoid indole alkaloid (*R*)-(–)-desprenylcarquinostatin A (**178**, Scheme 21a):^99^ In this case, a late synthetic intermediate of **178** was subjected to kinetic resolution by commercial lipase QLM,^35^ which proceeded with excellent enantioselectivity (*E* > 200) to afford the (*R*)-acetate and the (*S*)-alcohol in 98% and 97% *ee*, respectively. The synthesis was completed *via* oxidation to the quinone and, for the natural (*R*)-enantiomer, hydrolysis of the acetate. Lipase catalysis has also been employed in the kinetic resolution of vasicinone (**179**; lipase PS^35^, *E* > 200), sedamine (**69**, Fig. 2; porcine pancreatic lipase, *E* = 35), as well as lupinine (**180**; lipase AK,^35^ *E* = 24),^100^ and in the desymmetrisation of lobelanidine (**181**) with CAL-B, which afforded the (*S*)-monoacetate **182** in 70% yield and >98% *ee* (Scheme 21b).^101^ The latter compound was transformed into (–)-lobeline (**183**), a natural product with anti-addictive properties, by oxidation and ester hydrolysis.

A remarkable case of remote stereocentre discrimination in lipase-catalysed kinetic resolution has been reported by Fülöp and co-workers:^102^ The authors prepared both enantiomers of the tetrahydroisoquinoline alkaloid crispine A (**185**) *via* the open-chain analogue **184**, featuring a primary alcohol in δ-position of the chiral centre (Scheme 21c). Decanoylation of *rac*-**184** was found to be catalysed by lipase PS^35^ with surprisingly high enantioselectivity (*E* = 68), and by employing a two-step kinetic resolution protocol both the (*S*)-ester and the (*R*)-alcohol could be obtained in good optical purity (*ee* = 94% and 95%, respectively). Hydrolytic kinetic resolution of the racemic decanoate was also possible, but proceeded with somewhat lower enantioselectivity (*E* = 52). Conversion of the enantiomerically enriched alcohol **184** into crispine A was accomplished in a single step by treatment with thionyl chloride.

### 3.2 Monoamine oxidase in chemo-enzymatic deracemisation systems

**Scheme 22:**
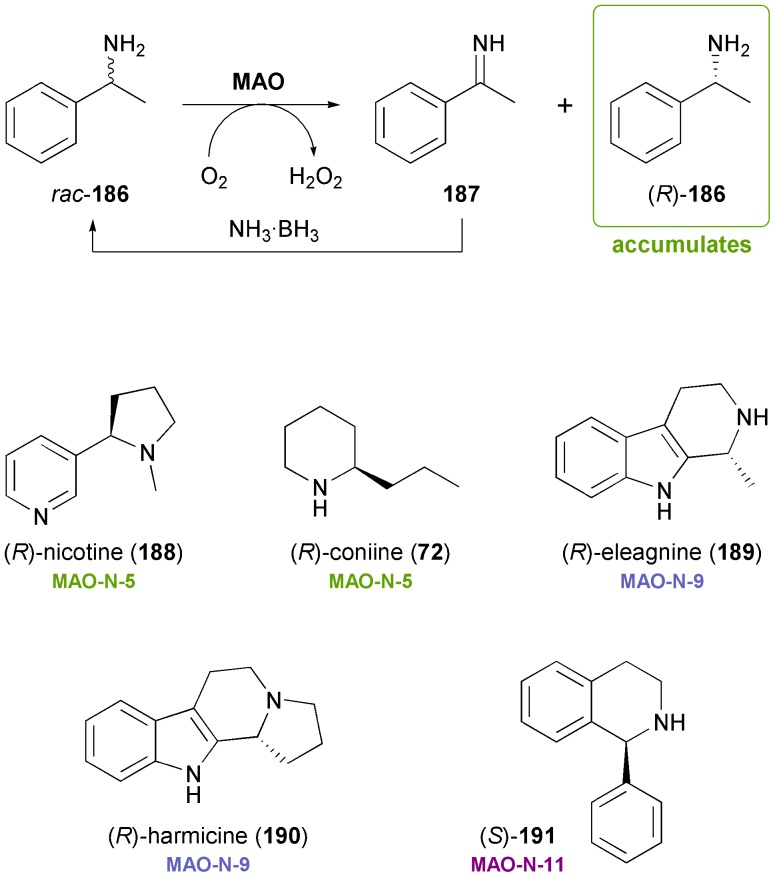
Chemo-enzymatic deracemisation of amines *via* MAO-catalysed oxidation illustrated with 1-phenylethylamine **186**, and examples of alkaloids that have been obtained employing engineered variants of MAO-N.

Monoamine oxidases (MAOs) are flavin-dependent enzymes catalysing the aerobic oxidation of amines to the corresponding imines or iminium ions. The one-pot combination of this reaction with chemical reduction of the imine leads–over several cycles of enantioselective oxidation and non-stereoselective reduction–to accumulation of the slower reacting amine enantiomer (Scheme 22). Over the last ten years, Nicholas Turner's research group has developed several highly enantioselective variants of MAO from *Aspergillus niger* (MAO-N), which have been engineered for the deracemisation of primary, secondary, and tertiary amines.^103^ Recent variants featuring a comparably broad substrate scope are interesting biocatalysts in the context of alkaloid synthesis,^104^ as has first been demonstrated by stereoinversion of (*S*)-nicotine (**188**) to the non-natural (*R*)-enantiomer by MAO-N-5 (Scheme 22).^105^ The same enzyme variant has also been found capable of deracemising crispine A (**185**, Scheme 21c), although in this case the reaction proceeded rather slowly.^106^ Further optimisation of the biocatalyst through directed evolution has led to the identification of a variant termed MAO-N-9, which displays exceptionally high activity (>5.5 kU/mg) towards crispine A.^107^ Very recent studies have demonstrated the broad applicability of MAO-N in the asymmetric synthesis of alkaloids:^108^ Variant MAO-N-9 was applied in the chemo-enzymatic preparation of the indole alkaloids (*R*)-eleagnine (**189**) and (*R*)-harmicine (**190**), while MAO-N-5 was used in the deracemisation of the hemlock neurotoxin coniine (**72**). In addition, a variant with opposite enantioselectivity (MAO-N-11) has been developed and applied in the synthesis of (*S*)-1-phenyltetrahydroisoquinoline (**191**), a building block for the urinary antispasmodic drug solifenacin.^108^

### 3.3 Oxidative kinetic resolution of benzylisoquinolines by berberine bridge enzyme

**Scheme 23:**
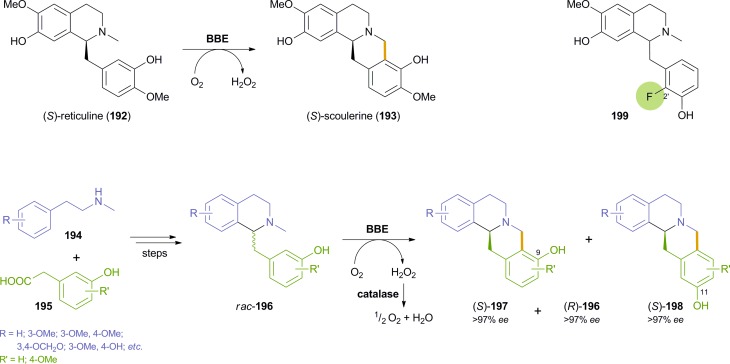
Oxidative cyclisation of (*S*)-reticuline **192** catalysed by BBE, application of BBE in the chemo-enzymatic asymmetric synthesis of (*S*)-berbines **197** and (*R*)-benzylisoquinolines **196** *via* kinetic resolution, and example of a substrate **199** that is cyclised to an 11-hydroxyberbine by BBE.

The berberine bridge enzyme (BBE, EC 1.21.3.3) is a bicovalent flavoenzyme found in various alkaloid-producing plants, most notably those of the *Papaveraceae* (poppy) and *Fumariaceae* (fumitory) families.^109^ It is a branch-point enzyme in benzylisoquinoline alkaloid biosynthesis and catalyses an extraordinary intramolecular oxidative carbon-carbon bond formation: At the expense of molecular oxygen as terminal electron acceptor, the natural substrate, (*S*)-reticuline (**192**), is oxidatively cyclised to give (*S*)-scoulerine (**193**) as sole product (Scheme 23)–a reaction that lacks any direct counterpart in synthetic chemistry. Studies on the mechanism^110^ and the structure^110a,111^ of BBE have provided deeper insight in the nature of this remarkable reaction, and the development of an efficient heterologous expression system (secretory expression in *Pichia pastoris*)^112^ enabled the production of the pure enzyme in amounts sufficient for detailed biocatalytic investigations.

Optimisation studies aimed at finding suitable reaction conditions for the conversion of non-natural benzylisoquinoline derivatives showed that BBE is a fairly stable biocatalyst able to work in the presence of various organic solvents and at relatively high substrate concentrations.^113^ Most importantly, however, BBE has been shown to convert several non-natural racemic substrates *rac*-**196**–bearing varied substituents on both aromatic rings–with excellent enantioselectivity (*E* > 200), thus affording both the berbine-type cyclisation products (*S*)-**197** and the remaining benzylisoquinolines (*R*)-**196** in optically pure form (*ee* > 97%; Scheme 23).^114^ In addition, the reactions proceeded with high selectivity towards the 9-hydroxyberbines **197**, while the regioisomeric products **198** were only formed in minor amounts (ratio **197**/**198** ≥ 86 : 14). Preparative transformations on 0.5 gram scale demonstrated the applicability of the BBE-catalysed kinetic resolution in the chemo-enzymatic synthesis of various benzylisoquinoline and berbine alkaloids, including the sedative and muscle-relaxing agent (*S*)-scoulerine (**193**), which was obtained in 7.4% yield over 9 steps.^114*b*^

The regiochemistry of the cyclisation was investigated in more detail in a follow-up study, which showed that the ratio of products **197** and **198** is influenced by the substitution pattern of the substrate, the pH of the reaction medium and the type and amount of co-solvents used. A complete switch in regioselectivity could be achieved by using substrates like compound **199**, in which the usual site of C–C coupling, position 2′, is blocked by a fluorine atom. Preparative-scale biotransformations of **199** and two related derivatives gave good to excellent yields (32–50%) of the (*S*)-11-hydroxyberbines and the remaining (*R*)-benzylisoquinolines, and even though the regioselectivity of the reaction had been changed, the enantioselectivity was fully conserved (*ee* > 97% for all compounds).^115^

### 3.4 Kinetic resolution of berbines by whole cells

**Scheme 24:**
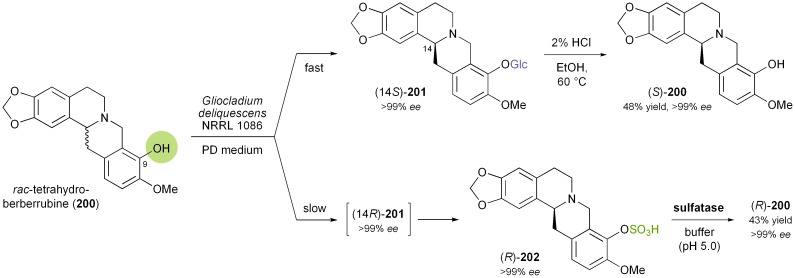
Kinetic resolution of tetrahydroberberrubine (*rac*-**200**) *via* kinetic glycosylation and enantioselective sulfation catalysed by *Gliocladium deliquescens*, and subsequent hydrolysis.

Another extraordinary example for a biocatalytic kinetic resolution of alkaloids has recently been reported by Ge *et al.*:^116^ The authors describe the kinetic resolution of tetrahydroberberrubine (*rac*-**200**) *via* kinetic glycosylation and enantioselective sulfation, both catalysed by whole cells of the fungus *Gliocladium deliquescens* NRRL 1086 (Scheme 24). Fermentation of **200** with the fungal culture in glucose-containing medium resulted in the formation of the diastereomeric β-glucosides (14*S*)- and (14*R*)-**201** (ratio 15 : 1), which could be separated by chromatography.^117^ Following the time course of the fermentation revealed that the (14*S*)-product was formed much faster than the (14*R*)-isomer, and that upon long-term incubation (120 h) the latter was converted further into the sulfate ester (*R*)-**202**. This sulfation reaction proved more selective than the glycosylation, as (14*S*)-**201** was not converted at all. Sulfate ester (*R*)-**202** was hydrolysed using commercially available sulfatase type H1 to give (*R*)-**200** in 43% overall yield and >99% *ee*. Likewise, the (14*S*)-glycoside **201** was subjected to acid-catalysed hydrolysis to afford (*S*)-**200** in 48% yield, also maintaining an *ee* of >99%. Testing the method on five closely related berbine alkaloids showed that the glycosylation only takes place when the hydroxyl group of the substrate is located in position 9 of the berbine scaffold.

## 4. Biocatalytic asymmetric key C–C and C–N bond formation

In the chemo-enzymatic syntheses discussed in the previous sections, the biocatalytic step appears either relatively early or at the very end of a longer sequence. However, enzymes can also take a more central role in alkaloid synthesis by catalysing key bond forming reactions in a highly stereoselective fashion. This strategic application of biocatalysis for chemically challenging reactions does not only open up novel routes towards the target compounds, it also often reduces the requirement for protective-group manipulations and thus leads to shorter and more practical syntheses.

### 4.1 Aldolases

**Scheme 25:**
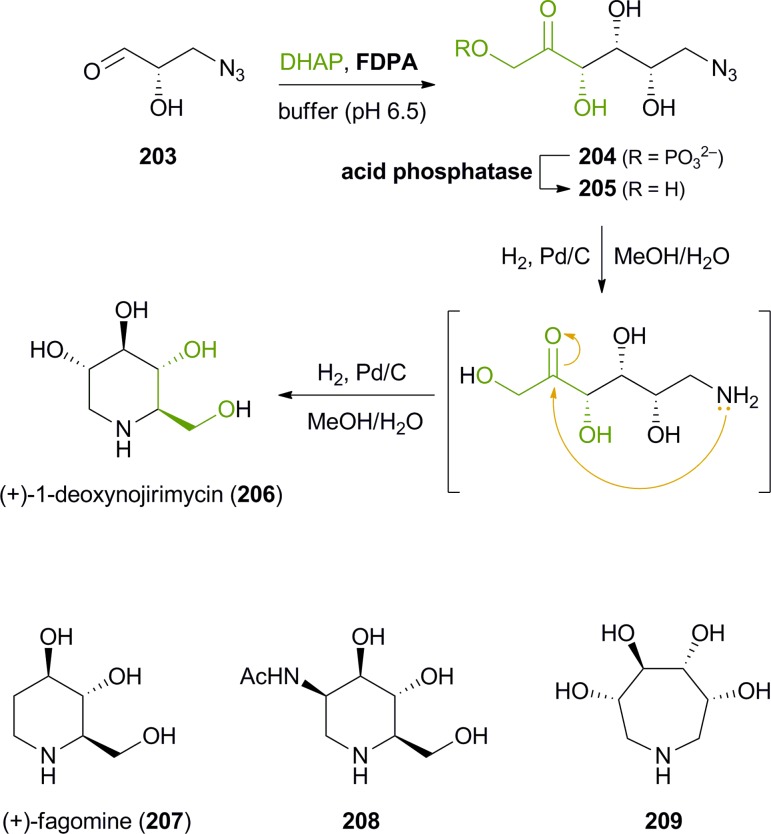
Chemo-enzymatic synthesis of (+)-1-deoxynojirimycin (**206**) *via* aldolase catalysis and intramolecular reductive amination, and examples of further iminocyclitols synthesised in a similar manner.

The synthesis of iminocyclitols (‘aza-sugars’) provides some excellent examples of the use of enzymes in key C–C bond forming reactions. Early contributions to the field have come from the research groups of Chi-Huey Wong and of Franz Effenberger, who employed d-fructose-1,6-diphosphate aldolase (FDPA) for highly stereoselective aldol reactions *en route* to (–)-d-1-deoxymannojirimycin (**103**, Fig. 3), (+)-d-1-deoxynojirimycin (**206**), and (+)-d-fagomine (**207**, Scheme 25).^118^ In these chemo-enzymatic syntheses, the aldolase catalyses the coupling of dihydroxyacetone phosphate (DHAP) with an aldehyde featuring an azide or Cbz-amine substituent. Upon catalytic hydrogenation of the dephosphorylated linear aldol product, a free amine is formed and the iminocylitol ring is closed by intramolecular reductive amination. The target compounds were obtained in overall yields ranging from 30% to 80%. Aza-analogues of *N*-acetylglucosamine and *N*-acetylmannosamine, *e.g.* **208** (Scheme 25), as well as derivatives featuring a phosphonic acid moiety or coupled to guanosine diphosphate have been prepared in a similar manner.^119^ Epimeric products have been synthesised using DHAP-dependent aldolases with different stereoselectivity, *e.g.* l-fuculose-1-phosphate aldolase (FucA) or l-rhamnulose-1-phosphate aldolase (RhuA).^120^ In addition, the scope of accessible products has been extended considerably by incorporating an enzyme-catalysed isomerisation of the linear sugar analogues (using *e.g.* glucose isomerase or fuculose isomerase) into the synthetic sequence. Thus, unusual 7-membembered iminocyclitols such as **209** have been prepared.^121^

In contrast to azidoaldehydes such as **203**, Cbz-protected amino aldehydes are rather poor substrates for DHAP-dependent aldolases, a fact that has been attributed to their low aqueous solubility. Espelt *et al.* have demonstrated that the use of emulsion systems can alleviate this problem and can thus lead to higher yields of the desired products.^122^ A more serious practical limitation of the above-mentioned aldolases is the requirement for expensive and unstable DHAP as a donor substrate. Although it has been demonstrated that *in situ* formation of DHAP from cheap precursors is possible,^123^ it would clearly be advantageous if the involvement of DHAP could be avoided completely. This is indeed possible using d-fructose-6-phosphate aldolase (FSA) from *E. coli*, an enzyme that is structurally related to transaldolases and accepts dihydroxyacetone and other non-phosphorylated ketones as donor substrates.^124^ Using this biocatalyst, the efficiency of chemo-enzymatic iminocyclitol synthesis has been considerably improved over the FDPA-based processes (d-fagomine, for instance, has been obtained in 51–65% isolated yield from dihydroxyacetone within two synthetic steps) and novel aza-sugars derived from hydroxyacetone and 1-hydroxy-2-butanone have become accessible.^125^

**Scheme 26:**
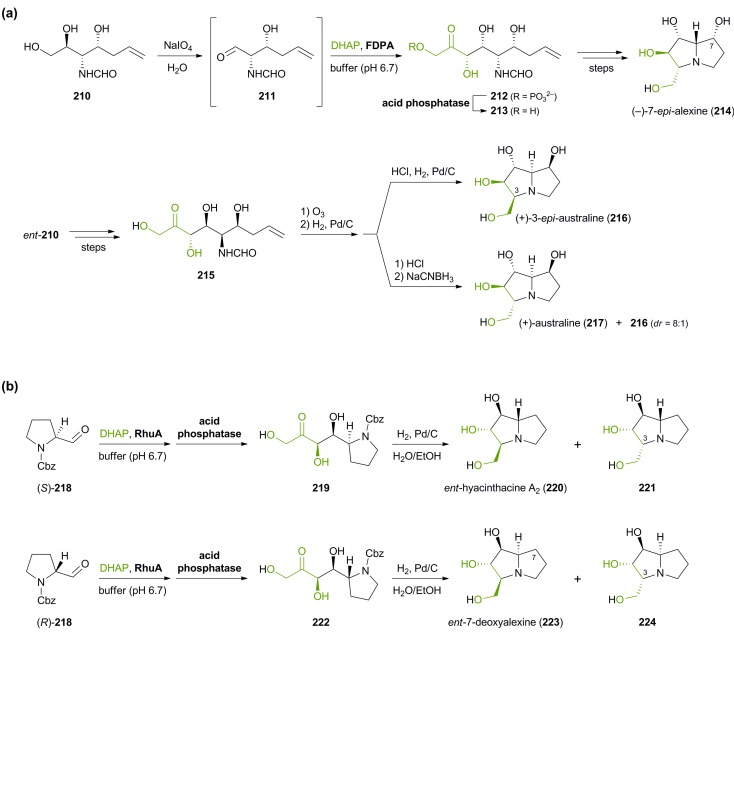
Aldolase-catalysed C–C bond formation in the synthesis of pyrrolizidine alkaloids: (a) chemo-enzymatic preparation of (–)-7-*epi*-alexine (**214**), (+)-3-*epi*-australine (**216**), and (+)-australine (**217**) using d-fructose-1,6-diphosphate aldolase (FDPA), and (b) chemo-enzymatic preparation of hyacinthacine isomers **220**, **221**, **223** and **224** using l-rhamnulose-1-phosphate aldolase (RhuA).

Besides the preparation of iminocyclitols, aldolases have also found application in the asymmetric synthesis of polyhydroxylated pyrrolizidine alkaloids: Romero & Wong have employed FDPA in the syntheses of (–)-7-*epi*-alexine (**214**), (+)-3-*epi*-australine (**216**), and (+)-australine (**217**, Scheme 26a).^126^ DHAP served as aldol donor in the biotransformation, while the aldol acceptor was the complex aldehyde **211**, generated *in situ* by oxidative cleavage of the corresponding vicinal diol **210** with NaIO_4_ in water. Divergence between australine and 3-*epi*-australine was achieved through choice of conditions in the final intramolecular reductive amination step: Catalytic hydrogenation afforded **216** as a single stereoisomer, while the use of sodium cyanoborohydride led to the formation of australine (**217**; albeit in an 8 : 1 mixture with **216**). The synthesis of structurally related pyrrolizidines, which represent stereoisomers of the naturally occurring alkaloids hyacinthacine A_1_ and A_2_, has recently been reported by Clapés and co-workers.^127^ In this case, Cbz-protected prolinal **218** served as acceptor substrate, contributing one ‘half’ of the pyrrolizidine core. Coupling of the prolinal derivative with DHAP catalysed by l-rhamnulose-1-phosphate aldolase (RhuA) from *E. coli* proceeded with excellent diastereoselectivity, but the following catalytic hydrogenation afforded mixtures of C3-epimeric products.^128^ Fortunately, the diastereomers proved separable by ion-exchange chromatography, allowing the isolation of the four hyacinthacine stereoisomers **220**, **221**, **223** and **224** in 9–23% yield from **218** (Scheme 26b). In a later study, further isomers and some closely related pyrrolizidines were prepared in the same manner, using an engineered variant of l-fuculose-1-phosphate aldolase (FucA) as biocatalyst.^129^ Finally, the concept has also been extended to indolizidines and quinolizidines, obtained *via* FucA- or RhuA-catalysis from 2-piperidyl-substituted aldehydes.^130^

### 4.2 Transaminases

**Scheme 27:**
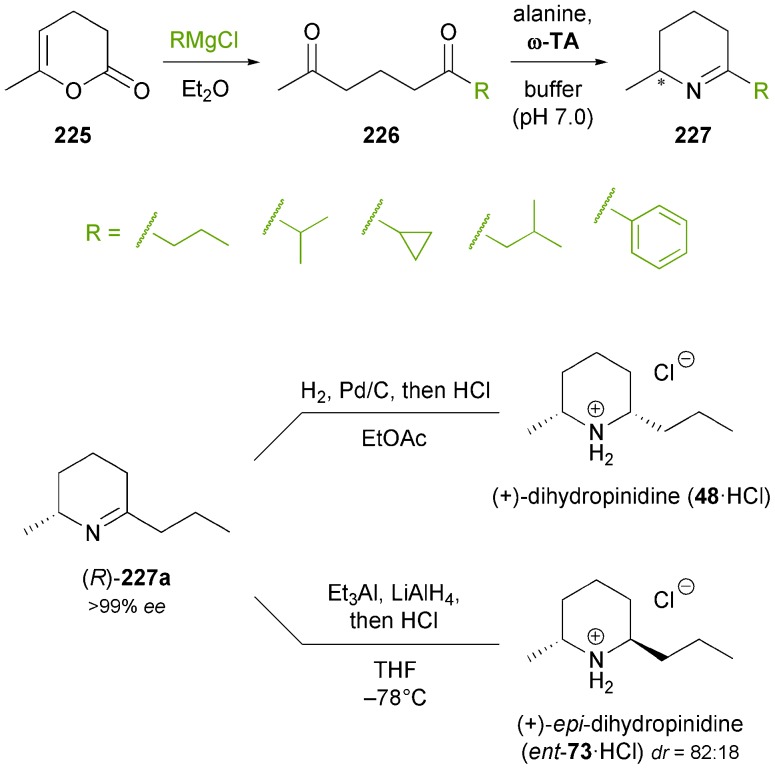
Regioselective asymmetric mono-amination of 1,5-diketones **226** catalysed by ω-transaminases, and its application to the chemo-enzymatic synthesis of dihydropinidine (**48**) and *epi*-dihydropinidin (*ent*-**73**).

Recent years have witnessed the ‘coming of age’ of ω-transaminases, which have been intensively studied as biocatalysts for the asymmetric synthesis or kinetic resolution of a wide range of chiral amines,^103b,131^ and which have also found industrial application.^132^ The prospect of gaining direct access to chiral primary amines from the corresponding ketones in high enantiomeric excess makes ω-transaminases also promising biocatalysts for the asymmetric synthesis of alkaloids, but their potential in this respect has only been started to be explored. Kroutil and co-workers have recently reported an application of ω-transaminases in the synthesis of piperidine alkaloids:^133^ The authors demonstrated that the reductive amination of δ-diketones **226** catalysed by various ω-transaminases proceeds exclusively at the less hindered carbonyl group (only in one case were minor amounts of the regioisomeric product detected). The amino ketones thus formed cyclised spontaneously to the corresponding cyclic imines, which upon catalytic hydrogenation afforded *cis*-2,6-disubstituted piperidines as single stereoisomers (Scheme 27). Since both (*R*)- and (*S*)-selective ω-transaminases could be employed in this chemo-enzymatic sequence, it provided access to both enantiomers of the final products. Thus, natural (+)-(2*R*,6*S*)-dihydropinidine (**48**) and its non-natural enantiomer were obtained in 65% and 72% overall yield, respectively, and only three steps from pyranone **225**. In a recent follow-up study, the authors have also succeeded in establishing a *trans*-selective reduction protocol (LiAlH_4_ reduction at low temperature in the presence of Et_3_Al as a Lewis acid), which provided *epi*-dihydropinidine (*ent*-**73**) in good, but not perfect diastereoselectivity (*dr* = 18 : 82 *syn*/*anti*).^134^ Furthermore, the transamination/hydrogenation sequence has recently been applied in the asymmetric synthesis of the fire ant venom alkaloid isosolenopsin, a longer-chain analogue of dihydropinidine.^135^

**Scheme 28:**
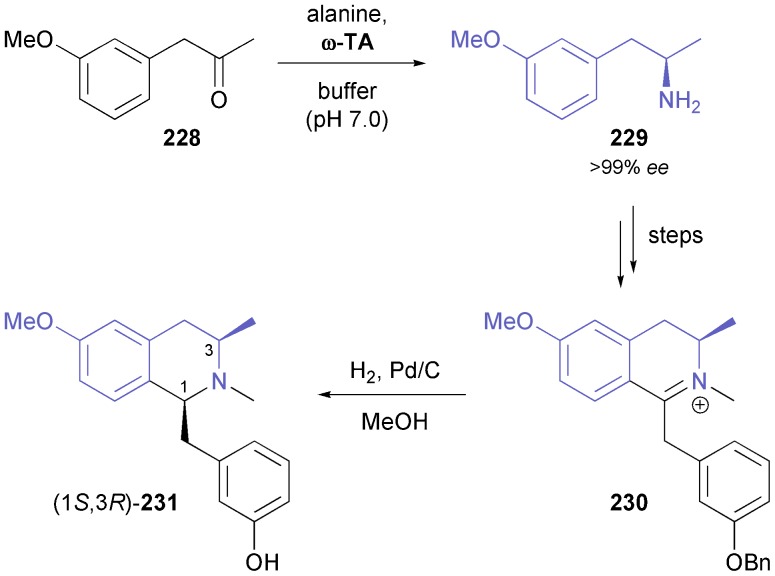
Synthesis of isoquinoline alkaloid **231** *via* a combination of enzymatic transamination and catalytic imine hydrogenation.

A combination of enzymatic transamination and subsequent imine reduction has also been used in the synthesis of the 1,3-disubstituted tetrahydroisoquinoline **231** and a second, structurally related compound (Scheme 28).^136^ Closure of the tetrahydroisoquinoline ring was achieved by a Bischler–Napieralski cyclisation, and catalytic hydrogenation again proceeded with perfect diastereoselectivity, affording (1*S*,3*R*)-**231** in 30% overall yield over six steps from ketone **228**.

### 4.3 Pictet–Spenglerases

**Scheme 29:**
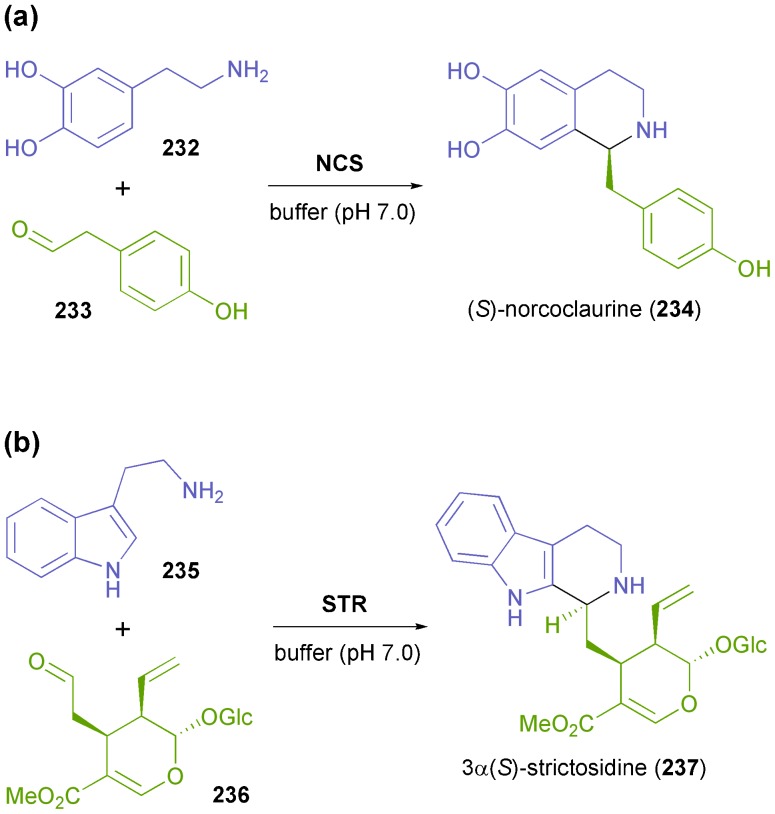
The natural reactions of (a) norcoclaurine synthase and (b) strictosidine synthase, which involve C–N bond formation and stereoselective C–C bond formation through an asymmetric Pictet–Spengler reaction.

In recent years, two C–C bond-forming enzymes involved in alkaloid biosynthesis have attracted increased attention from biocatalysis researchers: norcoclaurine synthase (NCS, EC 4.2.1.78) and strictosidine synthase (STR, EC 4.3.3.2) both catalyse asymmetric Pictet–Spengler reactions and are hence referred to as ‘Pictet–Spenglerases’.^137^ As such, they both join two relatively simple molecules–a 2-arylethylamine and an aldehyde–to form a nitrogen heterocycle with excellent stereo-chemical control of the resulting chiral centre (Scheme 29). Since asymmetric Pictet–Spengler reactions are still difficult to achieve with chemo-catalytic methods,^137^ NCS and STR offer the chance to establish novel, otherwise non-viable synthetic routes towards benzylisoquinoline and indole alkaloids.

#### 4.3.1 Norcoclaurine synthase

**Scheme 30:**
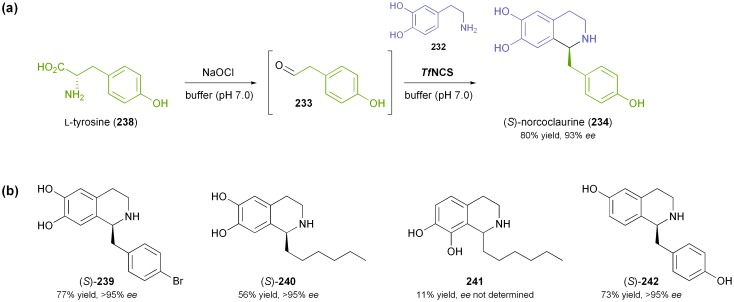
Application of norcoclaurine synthase in the asymmetric synthesis of tetrahydroisoquinolines: (a) one-pot chemo-enzymatic synthesis of (*S*)-norcoclaurine (**234**) using NCS from *Thalictrum flavum*, (b) non-natural tetrahydroisoquinolines **239–242** prepared using NCS from *Coptis japonica*.

Norcoclaurine synthase is a plant enzyme that catalyses the first committed step in the synthesis of benzylisoquinoline alkaloids–the condensation of dopamine (**232**) and 4-hydroxyphenylacetaldehyde (**233**) forming (*S*)-norcoclaurine (**234**) as product (Scheme 29a).^138^ The enzyme has first been isolated from plant tissue and partially characterised in 2001,^139^ and soon afterwards the cloning and heterologous expression in *E. coli* have been reported.^140^ Mechanistic studies^141^ and functional analysis^142^ followed, and soon the potential of NCS as a biocatalyst for asymmetric synthesis was recognised: Bonamore *et al.* have described the one-pot chemo-enzymatic synthesis of (*S*)-norcoclaurine (**234**) using NCS from *Thalictrum flavum* and *in situ* generation of **233** *via* NaOCl-oxidation of tyrosine (**238**, Scheme 30a).^143^ The two steps were carried out sequentially in phosphate buffer as reaction medium, and (*S*)-**234** was obtained in 81% yield and 93% *ee*. Later investigations by Pesnot *et al.* revealed that the choice of the right reaction setup is crucial for stereoselectivity, since a background activity of phosphate buffer was detected.^144^ The same authors have developed a fluorescamine-based screening assay which was used to elucidate the substrate spectrum of NCS from *Coptis japonica*.^145^ Variations of the substitution patterns of both reaction partners were tested, revealing a rather limited scope of amine derivatives, while more than 10 structurally diverse aldehydes were accepted. Preparative biotransformations afforded (*S*)-tetrahydroisoquinolines, including the non-natural compounds **239–242** (Scheme 30b), in good to excellent yield (56–99%) and with an excellent *ee* of >95%. Interestingly, using heptanal as substrate gave rise to both the expected *para*-coupling product (*S*)-**240** and the rather unusual *ortho*-isomer **241**.

The substrate scope of NCS from *Thalictrum flavum* has also been investigated recently, focusing on variation of the aldehyde.^146^ Fifteen non-natural derivatives were found to be accepted, but unfortunately the enantiomeric purity of the reaction products was not investigated.

#### 4.3.2 Strictosidine synthase

The second Pictet–Spenglerase with application in biocatalysis is strictosidine synthase. In fact, STR was the first Pictet–Spenglerase to be found in nature, isolated and characterised. Its natural reaction is the condensation of tryptamine (**235**) and the monoterpenoid aldehyde secologanin (**236**) to form 3α(*S*)-strictosidine (**237**, Scheme 29b), representing the first committed step in the synthesis of monoterpenoid indole alkaloids.^137^ The first preparative application of this biotransformation dates back to 1982, when Pfitzner & Zenk immobilised a partially purified STR obtained from *Catharanthus roseus* cell cultures on CNBr-activated sepharose and used the immobilised enzyme for the gram-scale synthesis of 3α(*S*)-strictosidine (**237**).^147^

**Scheme 31:**
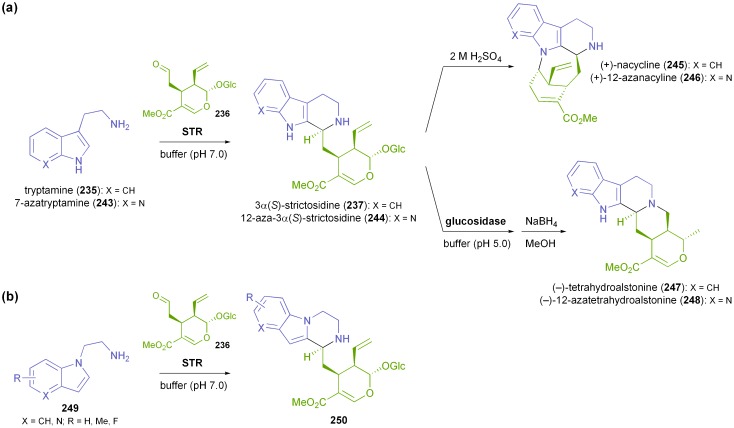
Biocatalytic application of strictosidine synthase: (a) chemo-enzymatic synthesis of (+)-nacycline (**245**), (–)-tetrahydroalstonine (**247**), and their aza-analogues, and (b) preparation of piperazino[1,2-*a*]indole alkaloids **250** using indole-1-ethanamine derivatives **249** as substrates.

More recently, the STRs from *Catharanthus roseus*, *Rauvolfia serpentina* and *Ophiorrhiza pumila* have been heterologously expressed and studied with respect to their substrate scope and their potential as biocatalysts, and the X-ray crystal structure of the *Rauvolfia* enzyme has been solved.^137,148^ In contrast to NCS, strictosidine synthases have generally been found to accept a broader range of amines, including the 1-oxa and 1-thia analogues of tryptamine, 7-azatryptamine (**243**), and various methylated or fluorinated derivatives.^137,148d,149^ The scope of aldehyde substrates was shown to be limited to secologanin (**236**) and some close analogues thereof for the two highly homologous STRs from *Catharanthus* and *Rauvolfia*.^149,150^ The *Ophiorrhiza* enzyme, on the other hand, accepts several simple aldehydes, *e.g.* hexanal or phenylacetaldehyde, as substrates, forming the corresponding tryptoline (tetrahydro-β-carboline) products in >98% *ee*.^151^ However, the observed rate constants (*k*_obs_) were in the low ‘per hour’-range, and the *K*_m_-values for the non-natural aldehydes were estimated to lie beyond the solubility limits of the investigated compounds. Because of these limitations, preparative-scale applications of strictosidine synthase have thus far been restricted to using secologanin (**236**) as aldehyde substrate. Nevertheless, structurally diverse products can be accessed by using chemo-enzymatic approaches, as has recently been demonstrated by Stöckigt, Yu, and co-workers:^152^ The authors describe the use of His-tagged *Rauvolfia* STR immobilised on a nickel-NTA column for the production of strictosidine (**237**) and its 12-aza-analogue **244**, both of which were converted further into related indole alkaloids. For instance, treatment with 2 M sulfuric acid resulted in the formation of (+)-nacycline (**245**) and (+)-12-azanacycline (**246**), while enzymatic deglycosylation^153^ followed by NaBH_4_ reduction provided (–)-tetrahydroalstonine (**247**) and the corresponding 12-aza-compound **248** (Scheme 31a). Overall yields of 18–60% (from **235**/**243**) were attained for these products.

Very recently, it has been discovered that *Rauvolfia* STR can also form heterocycles with an entirely different skeletal structure, as it can couple secologanin (**236**) with indole-1-ethanamine derivatives **249** to give piperazino[1,2-*a*]indole alkaloids **250** (Scheme 31b).^154^ Three products were isolated from small preparative-scale transformations (50 μmol substrate converted) and characterised by NMR, but no yields were reported. Still, this study demonstrates that also non-tryptoline alkaloids can be synthesised with the help of strictosidine synthase. The range of accessible products may be further expanded by using engineered STR variants,^155^ as has already been shown by studies *in planta*.^156^

## 5. Conclusions and outlook

**Fig. 7:**
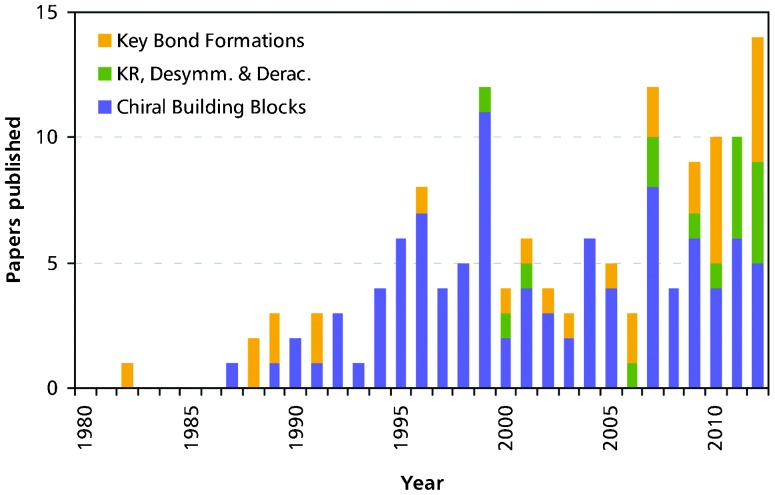
Chemo-enzymatic total syntheses of alkaloids published per year. The colours indicate the different chemo-enzymatic approaches as discussed in this review: (a) biocatalytic preparation of chiral building blocks (blue), (b) biocatalytic kinetic resolution, desymmetrisation, and deracemisation of alkaloids (green), and (c) chemo-enzymatic syntheses that use enzymes for key asymmetric C–C and/or C–N bond formation (orange).

Over the last decades, chemo-enzymatic alkaloid synthesis has developed into a prospering field of research that complements the established options for alkaloid production–isolation, semi-synthesis and total organic synthesis. For many years, research in the field has been dominated by chiral building block approaches relying mainly on lipases, esterases and toluene dioxygenase as biocatalysts, and due to its flexibility this research branch is still well represented today. However, the role of biocatalysis in the asymmetric synthesis of alkaloids has also been significantly extended in recent years: Kinetic resolution and deracemisation of entire alkaloids, as well as biocatalytic key C–C bond formation have gained importance (Fig. 7), and the scope of used enzymes has become a lot broader. An important factor in these developments is the exploitation of enzymes involved in alkaloid biosynthesis, such as norcoclaurine synthase, strictosidine synthase, and berberine bridge enzyme, which are able to carry out asymmetric transformations that are not yet possible by purely chemical means. Indeed, one third of the reports on chemo-enzymatic alkaloid syntheses published in the last three years (11 out of 33, 2010–2012) use enzymes from plant secondary metabolism.

Furthermore, the development of novel chemo-enzymatic strategies for alkaloid synthesis, in which biocatalysis takes a more central role, has led to shorter routes requiring less protective group chemistry than the classical building block approaches. This fact can be illustrated by comparing the ω-transaminase-based preparation of (+)-dihydropinidine (**48**, Scheme 27) recently reported by Simon *et al.* with earlier chemo-enzymatic syntheses of the same compound (Table 2). The transaminase route is not only the shortest one by far, it also gives a more than three times higher yield than the second-best alternative. In addition, this synthesis also compares favourably with purely chemical asymmetric syntheses of dihydropinidine.^133^

**Table 2:** Comparison of different chemo-enzymatic syntheses of (+)-dihydropinidine (**48**)

Article	Starting material	Steps	Biocatalytic step	Overall yield	Ref.
Momose *et al.* 1992	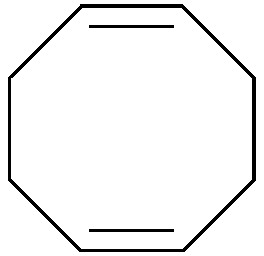	24	lipase-catalysed desymmetrisation	<1%^*a*^	45*a*
Chênevert & Dickman 1996	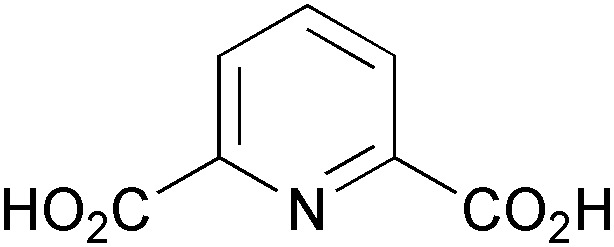	14	lipase-catalysed desymmetrisation	18%	48*b*
Yamauchi *et al.* 2004	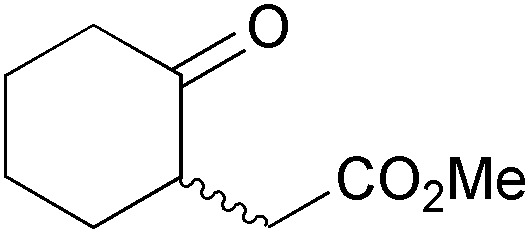	17	baker's yeast reduction	0.7%	159
Simon *et al.* 2012	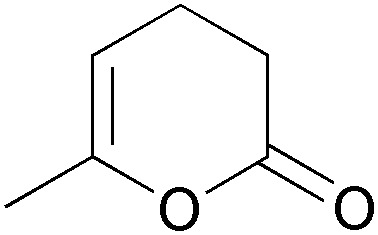	3	transaminase-catalysed reductive amination	65%	133

^*a*^Since no yields are reported for the last steps, an exact value can not be calculated.

The picture is similar when comparing different methods (Table 3) for the production of d-fagomine (**207**, Scheme 25), an iminosugar first isolated from buckwheat seeds in 1974,^157^ which is being discussed as a functional food ingredient due to its hypoglycaemic properties.^158^ Extraction of the alkaloid from natural sources gives only limited yields and requires at least three purification steps. State-of-the-art organic asymmetric syntheses provide **207** in up to 34% yield and require 6–9 steps; however, it must be taken into account that the reported ‘starting materials’ are not commercially available and therefore also need to be synthesised. The chemo-enzymatic route based on an asymmetric aldol reaction catalysed by d-fructose-6-phosphate aldolase (FSA) affords the target compound in only two steps and 51–65% yield from dihydroxyacetone and a Cbz-protected aminoaldehyde, which in turn can be easily prepared in 69% yield from 3-amino-1-propanol.

**Table 3:** Comparison of different production methods for d-fagomine (**207**)

Article	Starting material	Steps	Key steps	Overall yield	Ref.
Kato *et al.* 1997	leaves and roots of *Xanthocercis zambesiaca*	3^*a*^	extraction (50% aq. MeOH), ion exchange chromatography	*leaves*: 0.03%	160
				*roots*: 0.13%	
					
Asano *et al.* 2001	leaves of *Morus alba*	4^*a*^	extraction (50% aq. EtOH), ion exchange chromatography	0.02%	161
					
Kato *et al.* 2003	seeds of *Castanospermum australis*	3^*a*^	extraction (50% aq. MeOH), ion exchange chromatography	0.04%	162
					
Banba *et al.* 2001	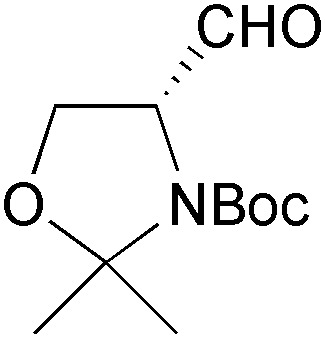	6	ring-closing metathesis, epoxidation, epoxide hydrolysis	12%	163
Takahata *et al.* 2003					
Kumari *et al.* 2009	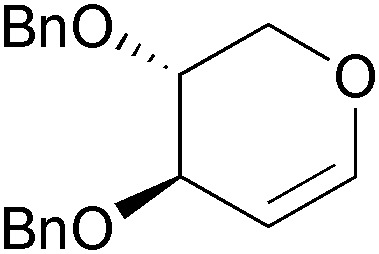	7	chloroamidation, intramolecular reductive amination	34%	164
Kim *et al.* 2011	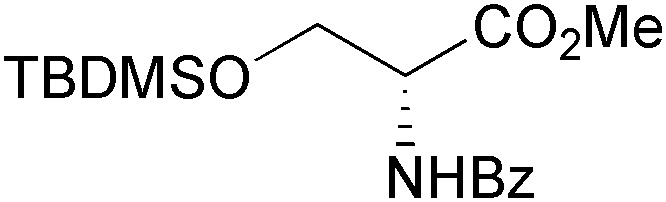	9	diastereoselective ketone reduction, Pd-catalysed intramolecular allylic substitution	19%	165
Kundu & Ghosh 2011	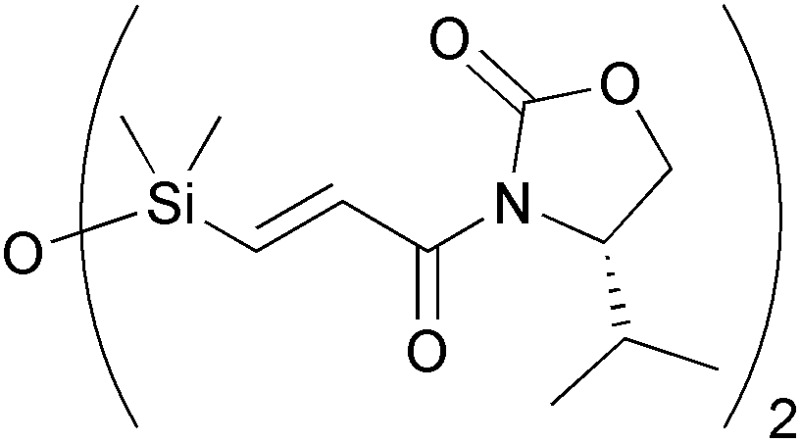	6	intramolecular reductive C–C coupling	11%	166
Castillo *et al.* 2006	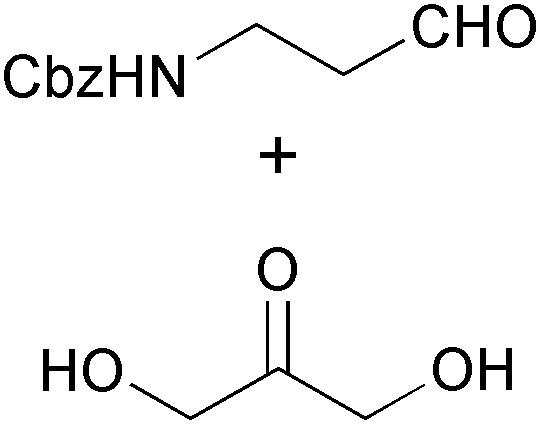	2	biocatalytic asymmetric aldol reaction, intramolecular reductive amination	Castillo: 51% Sugiyama: 65%	125*a*,*b*
Sugiyama *et al.* 2007					

^*a*^Purification steps.

Examples like these demonstrate that the strategic use of biocatalytic key transformations in chemo-enzymatic sequences can lead to truly practicable routes for the asymmetric total synthesis of alkaloids. The possibilities for further exploration of this concept in the future are vast: For instance, Pictet–Spenglerases other than norcoclaurine synthase and strictosidine synthase are known^137^ and might be applied in a biocatalytic context. Asymmetric reductive amination catalysed by ω-transaminases is likely to provide efficient access to further alkaloids or enable novel routes towards advanced intermediates in alkaloid synthesis. The broad applicability of chemo-enzymatic deracemisation using monoamine oxidase to complex, bulky amines–including several alkaloids–has just recently been demonstrated, and further developments in this field can be expected. Finally, the biosynthetic pathways that give rise to the incredible number and structural diversity of alkaloids found in nature represent an almost infinite source of novel enzymes that has just been started to be explored.

We believe that future developments along these lines–in combination with the modern possibilities of enzyme discovery and engineering,^167^ and recent efforts aimed at a better integration of biocatalytic and chemical transformations^168^–will continue to establish a more central role of biocatalysis in the asymmetric synthesis of alkaloids.

## Abbreviations

Ac: acetyl
AIBN: azobisisobutyronitrile
Alloc: allyloxycarbonyl
BBE: berberine bridge enzyme
Bn: benzyl
Boc: *tert*-butoxycarbonyl
BMIM: 1-butyl-3-methylimidazolium
Bz: benzoyl
CAL-B: lipase B from *Candida antarctica*
Cbz: carboxybenzyl
CDH: catechol dehydrogenase
CPDMO: cyclopentadecanone monooxygenase
CPMO: cyclopentanone monooxygenase
dba: dibenzylideneacetone
DEAD: diethyl azodicarboxylate
DHAP: dihydroxyacetone phosphate
DIBAL: diisobutylaluminium hydride
DMAP: 4-dimethylaminopyridine
DMF: *N*,*N*-dimethylformamide
DMS: dimethyl sulphide
dppe: 1,2-bis(diphenylphosphino)ethane
dppf: 1,1′-bis(diphenylphosphino)ferrocene
*dr*: diastereomeric ratio
*ee*: enantiomeric excess
FDPA: d-fructose-1,6-diphosphate aldolase
FucA: l-fuculose-1-phosphate aldolase
FSA: d-fructose-6-phosphate aldolase
*K*_m_: Michaelis constant
MAO: monoamine oxidase
MAO-N: monoamine oxidase from *Aspergillus niger*
MTBE: methyl *tert*-butyl ether
Ms: mesyl (methanesulfonyl)
NADPH: β-nicotinamide adenine dinucleotide phosphate
NCS: norcoclaurine synthase
NMM: *N*-methylmorpholine
NMO: *N*-methylmorpholine *N*-oxide
NMR: nuclear magnetic resonance
NTA: nitrilotriacetic acid
PLE: pig liver esterase
PPL: porcine pancreatic lipase
RhuA: l-rhamnulose-1-phosphate aldolase
S_N_: nucleophilic substitution
STR: strictosidine synthase
ω-TA: ω-transaminase
TBDMS: *tert*-butyldimethylsilyl
TBDPS: *tert*-butyldiphenylsilyl
TDO: toluene dioxygenase
TEMPO: 2,2,6,6-tetramethylpiperidine 1-oxyl
Tf: triflyl (trifluoromethanesulfonyl)
TFA: trifluoroacetic acid
THF: tetrahydrofuran
TMS: trimethylsilyl
Ts: tosyl (*p*-toluenesulfonyl)
